# The Wsp chemosensory system modulates c-di-GMP-dependent biofilm formation by integrating DSF quorum sensing through the WspR-RpfG complex in *Lysobacter*

**DOI:** 10.1038/s41522-022-00365-1

**Published:** 2022-12-16

**Authors:** Kangwen Xu, Limin Wang, Dan Xiong, Hongjun Chen, Xinru Tong, Xiaolong Shao, Tao Li, Guoliang Qian

**Affiliations:** 1 College of Plant Protection (Laboratory of Plant Immunity; Key Laboratory of Integrated Management of Crop Diseases and Pests), Nanjing Agricultural University, Nanjing, 210095 P.R. China; 2 Marine Agriculture Research Center, Tobacco Research Institute of Chinese Academy of Agricultural Sciences, Qingdao, 266101 P.R. China; 3 Shanghai Veterinary Research Institute, Chinese Academy of Agricultural Sciences, Shanghai, 200241 P.R. China

**Keywords:** Biofilms, Bacteria

## Abstract

The ubiquitous Wsp (wrinkly spreader phenotype) chemosensory system and DSF (diffusible signal factor) quorum sensing are two important chemically associated signaling systems that mediate bacterial communications between the host and environment. Although these two systems individually control biofilm formation in pathogenic bacteria via the ubiquitous second messenger c-di-GMP, their crosstalk mechanisms remain elusive. Here we present a scenario from the plant-beneficial and antifungal bacterium *Lysobacter enzymogenes* OH11, where biofilm formation favors the colonization of this bacterium in fungal hyphae. We found that the Wsp system regulated biofilm formation via WspR-mediated c-di-GMP signaling, whereas DSF system did not depend on the enzymatic activity of RpfG to regulate biofilm formation. We further found that WspR, a diguanylate cyclase (DGC) responsible for c-di-GMP synthesis, could directly bind to one of the DSF signaling components, RpfG, an active phosphodiesterase (PDE) responsible for c-di-GMP degradation. Thus, the WspR-RpfG complex represents a previously undiscovered molecular linker connecting the Wsp and DSF systems. Mechanistically, RpfG could function as an adaptor protein to bind and inhibit the DGC activity of unphosphorylated WspR independent of its PDE activity. Phosphorylation of WspR impaired its binding affinity to RpfG and also blocked the ability of RpfG to act as an adaptor protein, which enabled the Wsp system to regulate biofilm formation in a c-di-GMP-dependent manner by dynamically integrating the DSF system. Our findings demonstrated a previously uncharacterized mechanism of crosstalk between Wsp and DSF systems in plant-beneficial and antifungal bacteria.

## Introduction

Biofilms are structured communities of sessile microbial cells encapsulated by a self-secreted extracellular matrix composed of exopolysaccharides, proteins, and nucleic acids^1–3^. Previous reports have demonstrated that biofilms are essential for many pathogenic bacteria to colonize and further infect the hosts^4^. Moreover, pathogenic bacteria living in biofilms are more resistant to antibiotics and the host immune systems than planktonic cells, reinforcing the researchers’ focus on this bacterial lifestyle^5,6^. It is generally accepted that the regulation of pathogenic bacterial biofilm formation is a complex process involving multiple transcription factors and small-molecule chemicals, including the ubiquitous bacterial second messenger cyclic di-GMP (c-di-GMP)^7,8^. High levels of intracellular c-di-GMP commonly induce bacteria to switch from a planktonic state to a biofilm state, which has been well documented in several model pathogenic bacteria, *e*.*g*. the human pathogen *Pseudomonas aeruginosa*, *Salmonella enterica* and the plant pathogen *Xanthomonas campestris* pv. *campestris* (*Xcc*)^9–11^. It is well known that c-di-GMP is synthesized by diguanylate cyclases (DGC) containing a GGD/EEF motif that binds to two molecules of GTP as substrates, whereas it is degraded by phosphodiesterases (PDE) containing an EAL or HD-GYP motif to form one molecule of 5′-phosphoguanylyl-(3′-5′)-guanosine (pGpG) or two molecules of GMP^12,13^. The genomes of different bacteria usually encode multiple DGCs and PDEs, but only some of them affect biofilm formation under specific conditions^14,15^.

One of the c-di-GMP-dependent regulatory pathways for biofilm formation in pathogenic bacteria is the Wsp (wrinkly spreader phenotype) chemosensory system originally discovered in *P*. *aeruginosa*^9,16,17^. In this bacterium, the Wsp system is consisted of seven core proteins, namely, a chemoreceptor (WspA), two scaffolding proteins (WspB and WspD), a REC domain-containing histidine kinase (WspE), a methyltransferase (WspC), a REC domain-containing methylesterase (WspF), and a response regulator (WspR). WspR has a REC domain fused to the GGDEF domain, and phosphorylation of this REC domain enhances its DGC activity^17,18^. Previous studies found that inactivation of *wspF* in *P. aeruginosa*, which encodes an ortholog of E. coli CheB methylesterase, resulted in elevated intracellular c-di-GMP level and the formation of a wrinkly colony phenotype, thereby promoting biofilm formation^17,19,20^. In the *wspF* mutant background, this effect is caused by enhanced DGC activity of WspR through WspE phosphorylation^20,21^. Therefore, it was found that blockade of the methylesterase WspF artificially alters Wsp signaling from a locked state to an activated state by increasing intracellular c-di-GMP levels^20,21^. Under natural conditions, activation of the *P. aeruginosa* Wsp system responds to various environmental stimuli, including signals from solid surfaces or fatty acids^22^. Recent studies have shown that the Wsp system is sensitive to chemicals and mutations that perturb the cell envelope, particularly to stressors that affect the periplasmic space^23^. However, it remains unknown whether this chemosensory system can link with other chemical communication systems to regulate biofilm formation.

Diffusible signal factor (DSF)-dependent quorum sensing (QS) is another common bacterial chemical communication system^24,25^, originally discovered in the phytopathogenic bacterium *Xanthomonas campestris* pv. *campestris* (*Xcc*), and regulates biofilm formation in a c-di-GMP-dependent manner^26,27^. At the mechanistic level, the *rpf* gene cluster (regulation of pathogenicity factors) plays an important role in DSF synthesis and downstream signaling^28–30^. RpfF, a putative enoyl-CoA hydratase, is essential for the synthesis of DSF^31^, while the hybrid histidine kinase RpfC and the response regulator RpfG constitute a two-component system (TCS) involved in DSF signal sensing and transduction^32^. RpfG has an N-terminal REC domain fused to a C-terminal HD-GYP domain that degrades c-di-GMP^33^. At low cell density, RpfC is unphosphorylated and forms a protein complex with RpfF to limit DSF production^34^. At high cell density, accumulation of extracellular DSF triggers autophosphorylation of RpfC followed by phosphate transfer to RpfG, which stimulates the PDE activity of RpfG to trigger c-di-GMP degradation, thereby reducing biofilm formation^34,35^.

*Lysobacter enzymogenes* is a member of the soil microbiome and acts as a biocontrol agent capable of protecting a variety of crops from fungal infection^36,37^. *L. enzymogenes* can secret abundant lyases and an antifungal antibiotic termed heat-stable antifungal factor (HSAF) to attach and colonize fungal hyphae to inhibit fungal growth^38–40^. In previous works, we and collaborators have shown that both Wsp and DSF systems in *L. enzymogenes* regulate HSAF production under nutrient-limiting conditions^41,42^. The Wsp system regulates the HSAF biosynthesis through c-di-GMP signaling involving the WspR-CdgL binary complex, where WspR and CdgL act as DGC and c-di-GMP binding protein, respectively^42^. Phosphorylation of WspR activates its DGC activity and impairs WspR-CdgL binding affinity, thereby contributing to the accumulation of the c-di-GMP-bound CdgL, which in turn promotes the disassociation of the CdgL-LysR complex and reduces the apparent affinity of LysR to the promoter region upstream of the HSAF biosynthesis operon^42–44^. The DSF system regulates HSAF biosynthesis via c-di-GMP-independent signaling comprising RpfC, RpfF, RpfG and three hybrid TCS proteins (HtsH1, HtsH2, and HtsH3)^41^. RpfG is involved in HSAF biosynthesis by interacting with three hybrid TCS proteins that regulate the expression of HSAF synthesis genes through phosphorylation^41^. Moreover, inhibition of HSAF biosynthesis at elevated c-di-GMP levels also requires a unique c-di-GMP signaling pathway, comprising DGC LchD, PDE LchP, and the c-di-GMP-binding transcription factor Clp. In this way, LchD, LchP, and Clp seem to form a triple complex to maintain local c-di-GMP signaling^45,46^.

The aim of this study is to understand whether both the Wsp and DSF system are involved in c-di-GMP-dependent biofilm formation in the plant-beneficial *L. enzymogenes*. By using *L. enzymogenes* OH11 as a working model, we showed that both the Wsp and DSF system play key roles in regulating biofilm formation, the former via c-di-GMP but the latter does not appear to do so. Notably, we found that WspR and RpfG formed a WspR-RpfG complex linking the Wsp and DSF systems. Notably, our results showed that RpfG functions as an adaptor protein that binds and inhibits the DGC activity of WspR independently of its PDE activity. Thus, the WspR-RpfG complex represented a previously undiscovered molecular linker that enables a widespread Wsp system to control c-di-GMP-dependent biofilm formation by integrating with the bacterial DSF system.

## Results

### Activation of the Wsp system stimulates biofilm formation depending on c-di-GMP levels

**Fig. 1:**
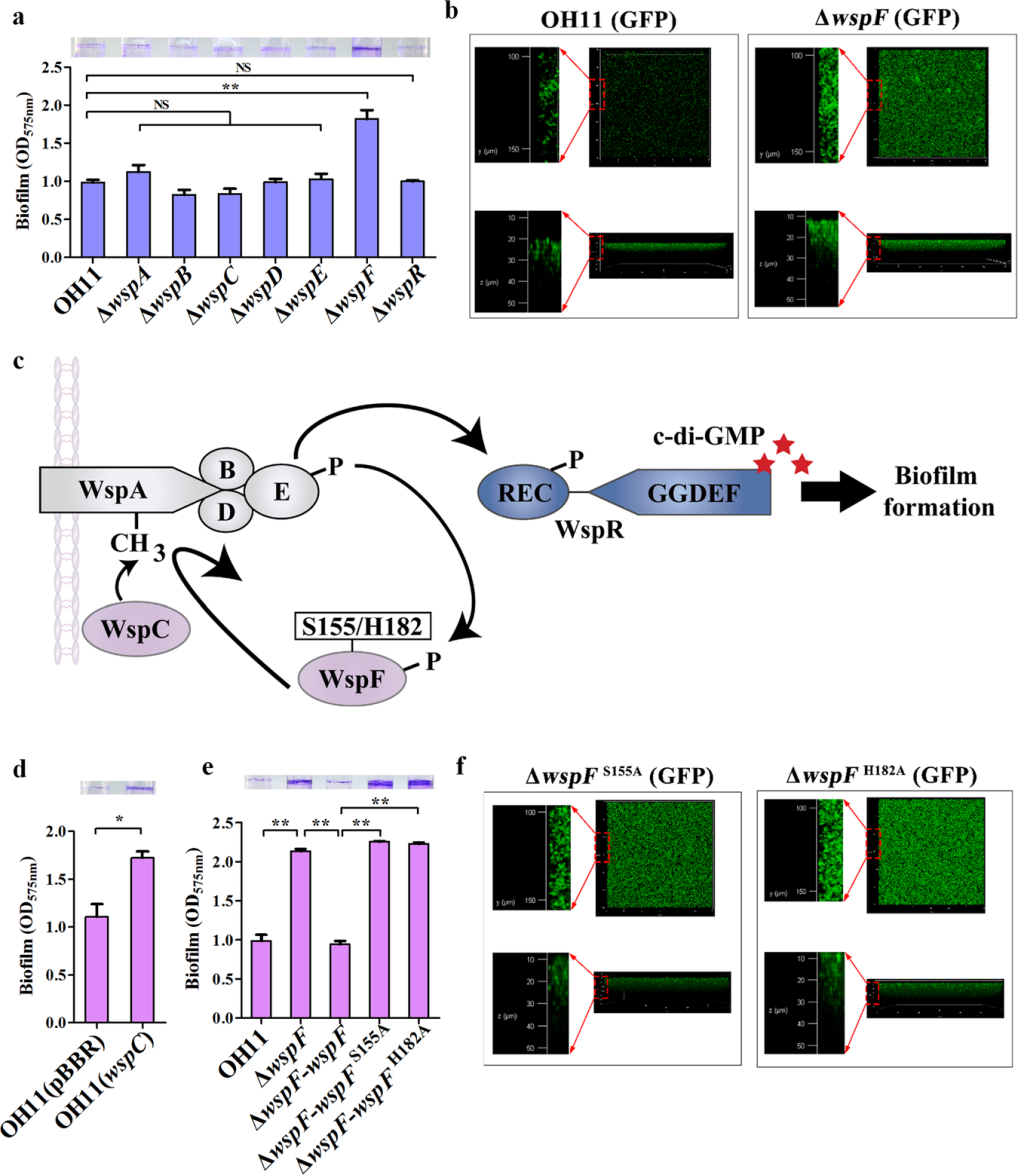
Activation of the Wsp signaling system promotes biofilm formation in L. enzymogenes. **a** Effects of all Wsp signaling components on biofilm biomass through individual mutations in their respective encoding genes. Crystal violet (CV)-stained tubes were diluted with ethanol-acetone and OD_575_ values measured to quantify biofilm biomass. On top, representative images of CV-stained biofilms were shown. **b** Confocal laser scanning microscopy (CLSM) of biofilms formed by wild-type and *wspF* mutant. CLSM was performed to observe the three-dimensional structure of biofilms. Images were acquired after 24 h using a 20× objective for green fluorescent protein (GFP)-labelled cells grown on chamber-covered glass slides. The upper part of the black box represents the top surfaces of the three-dimensional biofilm structure (y and x axes), while the lower part of the black box shows the side surfaces of the three-dimensional biofilm structure (z and x axes). Red circles indicate image magnification. **c** Schematic showing the Wsp signaling system for regulating c-di-GMP production in *P*. *aeruginosa*^16^. **d** Overexpression of *wspC* promoted biofilm biomass. **e** The enzymatic activity of WspF is crucial for regulating biofilm formation revealed by point mutations (S155A or H182A) of two predicated enzymatically active residues, S155 and H182. **f** Three-dimensional biofilm structures formed by *L. enzymogenes* carrying *wspF* variant (*wspF*^S155A^ or *wspF*^H182A^) detected by CLSM. OH11, wild type; Δ*wspA*-Δ*wspR*, in-frame deletion mutant of each corresponding *wsp* gene; Δ*wspF*-*wspF*, Δ*wspF*-*wspF*^S155A^ or Δ*wspF*-*wspF*^H182A^ indicate native *wspF*, *wspF*^S155A^ or *wspF*^H182A^ was chromosomally inserted into the *wspF* mutant; OH11 (GFP) or Δ*wspF* (GFP) represents wild-type OH11 and Δ*wspF* carrying a plasmid-borne GFP; *wspF*^S155A^ (GFP) and *wspF*^H182A^ (GFP) represent Δ*wspF*-*wspF*^S155A^ or Δ*wspF*-*wspF*^H182A^ carrying a plasmid-borne GFP. Statistical comparisons were performed using one-way ANOVA of GraphPad software (GraphPad, La Jolla, CA). In panels **a**, **d** and **e**, mean data ± SD from three experiments were shown, *P < 0.05; **P < 0.01. NS means not significant.

To test whether the Wsp system is involved in biofilm formation in *L. enzymogenes* under nutrient-rich conditions triggered by LB medium, we examined the biofilm biomass (bacteria and associated extracellular matrix) of seven laboratory-available Wsp mutants (Δ*wspA*, Δ*wspB*, Δ*wspC*, Δ*wspD*, Δ*wspE*, Δ*wspF* and Δ*wspR*) in polyethylene tubes using the crystal violet (CV) staining approach. We found that only the *wspF* mutant showed a significantly enhanced biofilm biomass compared to wild-type OH11 (Fig. 1a). To validate this result, we performed an additional assay using Confocal Laser Scanning Microscopy (CLSM). First, we generated a GFP-labelled *wspF* mutant or wild type by introducing the plasmid pBBRMCS-5-*gfp*. After static incubation in chambered coverslips for 24 h, GFP-tagged *wspF* mutants formed a structured biofilm in which bacteria were densely packed and organized, forming large aggregates extending across the entire surface. Under similar test conditions, GFP-tagged wild-type OH11 cells produced smaller dispersed biofilms that were far less organized than those produced by the *wspF* mutant (Fig. 1b). We compared the growth ability of wild type and *wspF* mutant in LB broth and found the cell density of *wspF* mutant was very close to that of wild-type OH11 after 24 h of incubation (Supplementary Fig. 1). At this time, biofilms of the two mutants, as well as wild-type OH11 were assayed. Together, these results suggested that WspF negatively regulated biofilm maintenance in *L*. *enzymogenes*.

Since WspF is predicted to be a methylesterase, we hypothesized that the *wspF* null mutation might accelerate the methylation of WspA, an MCP-like protein, and result in activation of the Wsp system to promote biofilm formation in *L. enzymogenes* (Fig. 1c), which has been demonstrated in *P. aeruginosa*^9^. To test this hypothesis, we first overexpressed the methyltransferase WspC in wild-type OH11, which indeed confirmed our hypothesis (Fig. 1d). In the second assay, we chromosomally altered two predicted enzymatically active site residues S155 and H182 of WspF to alanine (A) by double-crossover homologous recombination, and showed that, like the *wspF* mutant, each substitution significantly increased biofilm biomass (Fig. 1e), which was further confirmed by the CLSM method (Fig. 1f). Collectively, the above findings suggested that artificial activation of the Wsp system via *wspF* mutation promoted biofilm formation in *L*. *enzymogenes*.

**Fig. 2:**
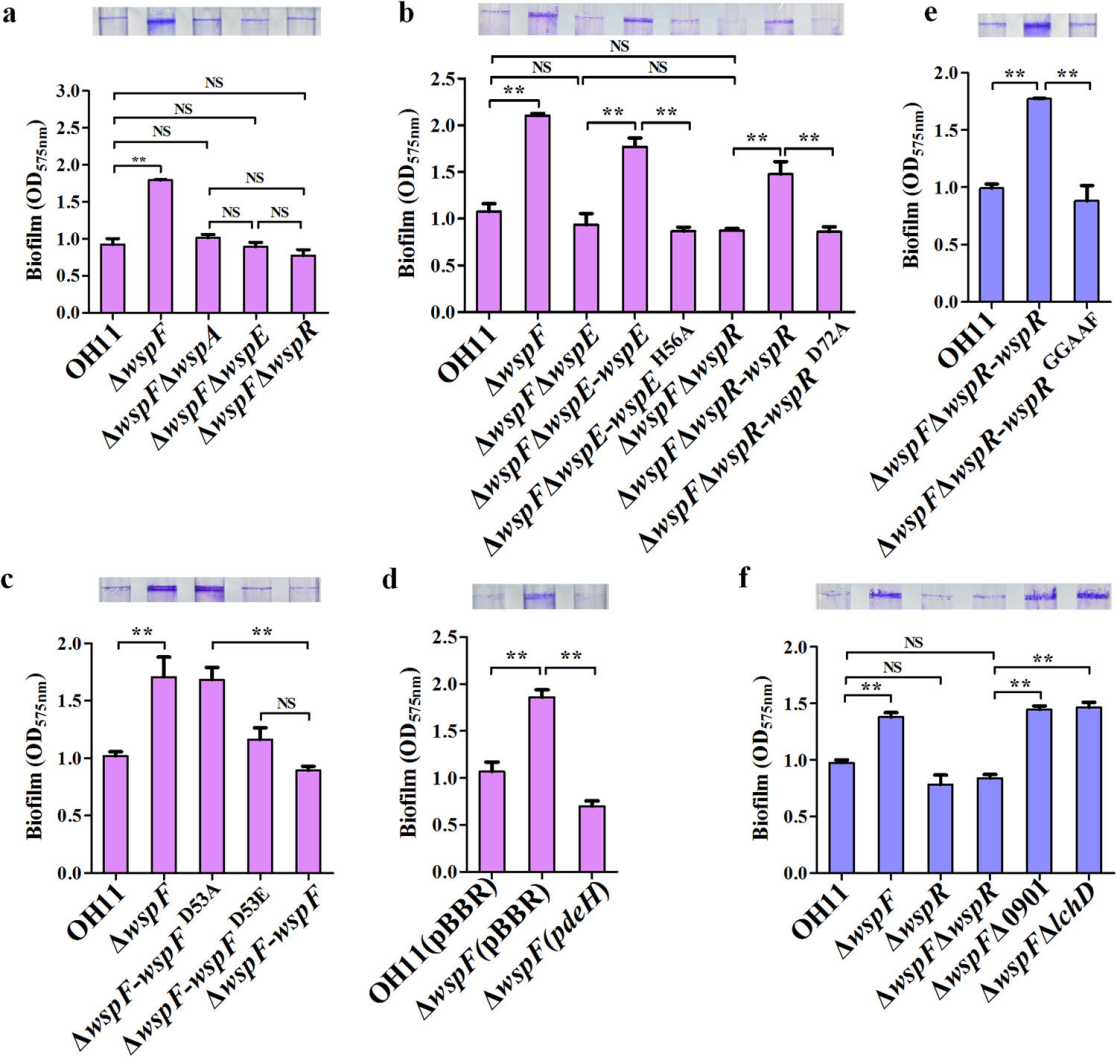
Wsp signaling system regulates biofilm formation in response to c-di-GMP. **a** Effects of several selected Wsp signaling components on biofilm biomass through combinatorial mutations of the respective encoding genes. Δ*wspF*Δ*wspA*, Δ*wspF*Δ*wspE* or Δ*wspF*Δ*wspR* represent double mutations. **b** Involvement of the key WspE autokinase residue (H56) or WspR phosphor-accepting residue (D72) in biofilm formation. Δ*wspF*Δ*wspE*-*wspE* and Δ*wspF*Δ*wspE*-*wspE*^H56A^ represent native *wspE* or its variant (*wspE*^H56A^) that were inserted into the chromosome of Δ*wspF*Δ*wspE* double mutant; Δ*wspF*Δ*wspR*-*wspR* and Δ*wspF*Δ*wspR*-*wspR*^D72A^ represent the native *wspR* and its variant (*wspR*^D72A^) that were individually inserted into the chromosome of Δ*wspF*Δ*wspR*. **c** The involvement of key WspF phosphor-receiving residue on biofilm biomass. Δ*wspF*-*wspF*^D53A^ and Δ*wspF*-*wspF*^D53E^ indicate the *wspF* variation (substitution of phosphor-receiving residue D53 to A53 or E53, respectively), which was individually inserted into the chromosome of *wspF* mutant. **d** The role of c-di-GMP concentration in biofilm biomass. Δ*wspF* (*pdeH*) denotes Δ*wspF* carrying plasmid-borne *pdeH* driven by a constitutive promoter of the vector, which produces active c-di-GMP PDE from E. coli. Δ*wspF* (pBBR) indicates Δ*wspF* carrying an empty vector. **e** The role of key GGEEF motif of WspR in biofilm biomass. Δ*wspF*Δ*wspR*-*wspR*^GGAAF^ represents a *wsp*R variant by replacing of GGEEF motif with GGAAF, which was inserted into the chromosome of Δ*wspF*Δ*wspR*. **f** WspR, but not the other two potential DGCs (Δ0901 and Δ*lchD*), mediates WspF signaling in biofilm biomass. Two delta symbols indicate gene double mutants. Statistical comparisons were performed using one-way ANOVA of GraphPad software (GraphPad, La Jolla, CA). In all assays, mean data ± SD from three experiments were shown, **P < 0.01. NS means not significant.

To validate the above findings, we further performed a series of genetic assays. First, we separately inactivated three genes encoding the core components of Wsp (*wspA*, *wspE* and *wspR*) in the *wspF* mutant background by in-frame deletions, and found that blocking each of them in the *wspF* mutant enabled biofilm biomass to return to the wild-type level (Fig. 2a). Second, in the *wspF* mutant, we chromosomally replaced the autophosphorylation residue H56 of WspE with A56 by means of double-crossover homologous recombination to prevent its autophosphorylation, and the results showed that the point mutant strain produced significantly reduced biofilm biomass (Fig. 2b). Using the same approach, we mutated the phosphorylation residue D72 of WspR to A72 in the *wspF* mutant to disrupt the transfer of the phosphate group from WspE. This step also significantly reduced biofilm biomass (Fig. 2b). Finally, we replaced the phosphorylation residue D53 of WspF in the chromosome with A53 and E53, respectively, in wild-type OH11. Replacing D53 with A53 was designed to prevent the transfer of phosphate group from WspE, which was expected to promote biofilm formation, whereas the replacement of D53 with E53 was aimed to artificially mimic WspF phosphorylation, and this is expected to inhibit biofilm formation. Indeed, the results were consistent with the above expectations (Fig. 2c). These additional genetic evidences doubly confirmed that activation of the Wsp system stimulated the biofilm biomass in *L. enzymogenes*.

To understand whether the functional output of the Wsp system in *L. enzymogenes* is directly related to the WspR-dependent c-di-GMP signaling that has been addressed in *P. aeruginosa*^9^, we carried out the following assays. First, we found that heterologous expression of the well-characterized c-di-GMP degrading enzyme gene *pdeH* from E. coli in *wspF* mutant restored its biofilm biomass to a wild-type level (Fig. 2d), indicating that the Wsp system works by increasing the intracellular c-di-GMP concentration in *L. enzymogenes*. To test whether WspR is involved in this process, we replaced the enzymatically active GGEEF motif of WspR in the chromosome with GGAAF in *wspF* mutant. The result showed that this residue substitution inhibited biofilm biomass (Fig. 2e). Meanwhile, overexpression of *wspR* enhanced biofilm formation (Supplementary Fig. 2). As a control, overexpression of *lchD*, another validated DGC gene^36^, did not show this function (Supplementary Fig. 2). Next, we deleted *wspR* in-frame in the *wspF* mutant, which restored biofilm biomass to wild-type level, while individual in-frame mutation of two additional DGC genes containing a GGD/EEF motif (*Le*0901 and *lchD*)^36^ in the *wspF* mutant did not exhibit this effect (Fig. 2f). Together, these results revealed that the Wsp system activated biofilm formation, depending on the DGC activity of WspR.

To validate above findings, we compared intracellular c-di-GMP concentrations in Δ*wspF*, Δ*wspF*Δ*wspR*, Δ*wspF*Δ*wspR*-*wspR* and Δ*wspF*Δ*wspR*-*wspR*^GGAAF^ using liquid chromatography-tandem mass spectrometry (LC-MS/MS). The result showed that the total c-di-GMP level of all tested strains remained consistent with the wild type (Supplementary Fig. 3). These results indicated that WspF most likely regulated biofilm formation through local c-di-GMP signaling in *L. enzymogenes*. To further confirm this, we carried out two additional assays. First, we determined the total c-di-GMP levels of *lchD-* and *wspR*-overexpressing strains by using LC-MS/MS. The results showed that *lchD* overexpression modestly increased the total intracellular c-di-GMP content compared with wild-type OH11 carrying an empty vector, whereas *wspR* overexpression did not change the total c-di-GMP content (Supplementary Fig. 4). Second, we also determined the total c-di-GMP level of *wspF*0901 and *wspFlchD* double mutants. The results showed that the content of total c-di-GMP in the *wspF*0901 and *wspFlchD* double mutants were consistent with the *wspFwspR* double mutant (Supplementary Fig. 5). Taken together, these results supported the conclusion that WspF may regulate biofilm formation through WspR-dependent local c-di-GMP signaling.

### The DSF system represses biofilm formation independently of the PDE activity of RpfG

**Fig. 3:**
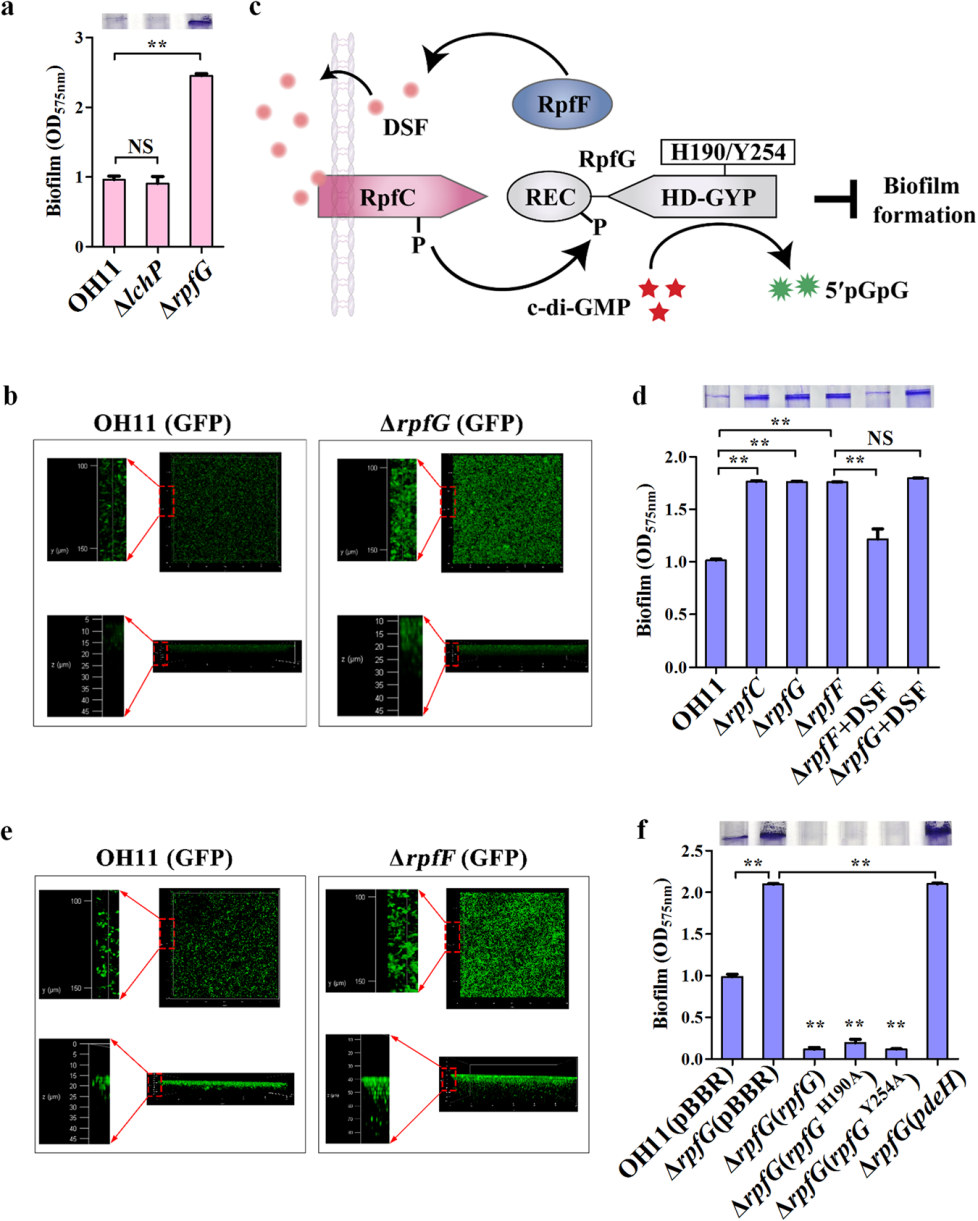
The DSF system inhibits biofilm formation independently of the PDE activity of RpfG in L. enzymogenes. **a** RpfG, but not LchP, negatively regulates biofilm formation. **b** CLSM of biofilm formed by wild-type and *rpfG* mutants. The upper part of the black box represents top surfaces (y and x axes) of the three-dimensional biofilm structures, while the lower part of the black box shows the side surfaces (z and x axes) of the three-dimensional biofilm structure. Red circles indicate image magnification. **c** Schematic showing the DSF system for regulating c-di-GMP production in *Xcc*. **d** Effect of all DSF system components on biofilm biomass through individual mutations in their respective encoding genes. Δ*rpfF* + DSF and Δ*rpfG* + DSF show the effect of adding 10 μM DSF on biofilm formation. **e** CLSM of biofilm formed by wild-type and *rpfF* mutant. **f** Involvement of key enzymatically active residues of RpfG in biofilm biomass. Δ*rpfG* (*rpfG*), Δ*rpfG* (*rpfG*^H190A^) or Δ*rpfG* (*rpfG*^Y254A^) indicates Δ*rpfG* carrying a plasmid-borne native *rpfG* or its variants with point mutations. Δ*rpfG* (*pdeH*) indicates Δ*rpfG* carrying a plasmid-borne *pdeH* driven by a constitutive promoter of the vector. Statistical comparisons were performed using one-way ANOVA of GraphPad software (GraphPad, La Jolla, CA). In panels **a**, **d** and **f**, mean data ± SD from three experiments were shown, **P < 0.01. NS means not significant.

To investigate whether the DSF system is also involved in c-di-GMP-dependent biofilm formation in *L. enzymogenes*, we examined the biofilm biomass of *rpfG* mutant encoding a functional PDE of this system, as previously described^41^. CV staining assay clearly showed that mutation of wild type *rpfG* significantly enhanced biofilm biomass (Fig. 3a). However, mutation of another experimentally validated PDE gene^45^, *lchP*, did not show this effect (Fig. 3a). The CLSM assay further validated the involvement of RpfG in inhibiting biofilm formation (Fig. 3b). Likewise, we compared the growth ability of wild-type OH11 and *rpfG* mutant in LB broth and found the cell density of *rpfG* mutant was very close to that of wild-type OH11 after 24 h of incubation (Supplementary Fig. 6).

Since RpfG is a core member of the DSF system (Fig. 3c), it is reasonable to speculate that the DSF system is likely involved in biofilm regulation in *L. enzymogenes*. To test this, we examined the biofilm biomass of *rpfC*, *rpfF* and *rpfG* mutants. CV staining assays showed that both mutants significantly promoted biofilm biomass (Fig. 3d). In *Xcc*, RpfF is a putative enoyl-CoA hydratase responsible for DSF synthesis^31^. Mutation of *rpfF* caused a complete defect in DSF production, thereby blocking the DSF signaling pathway. Furthermore, RpfG is a downstream component of DSF signaling^32^. In the *rpfG* mutant, the intact DSF signaling pathway is inactivated while RpfF remained active in producing DSF. To test whether the same mechanism exists in *L*. *enzymogenes*, we examined the biofilm biomass of *rpfF* and *rpfG* mutants in the presence of commercial DSF standard (10 μM). As expected, supplementation of external DSF to *rpfF* mutant culture nearly restored biofilm biomass to wild-type level, whereas no such phenotype rescue could be observed when DSF was added to *rpfG* mutant culture (Fig. 3d). In addition, we found that biofilms formed by GFP-labelled *rpfF* mutant was densely organized over the entire surface, as detected by the CLSM approach, just like the *rpfG* mutant (Fig. 3e). These findings suggested that an RpfG-dependent DSF system is essential for biofilm formation in *L. enzymogenes*. To test whether the PDE activity of RpfG is involved in this regulation, we tested RpfG^H190A^ and RpfG^Y254A^, two inactive variants of RpfG, as previously reported^41^. Introducing native plasmid-borne *rpfG* into *rpfG* mutant did dramatically reduce biofilm biomass compared to mutant expressing an empty vector (Fig. 3f). To our surprise, *rpfG* mutant expressing the plasmid-borne *rpfG*^H190A^ or *rpfG*^Y254A^ still inhibited biofilm formation (Fig. 3f). In support of this result, heterologous expression of another known PDE gene, *pdeH*, in the *rpfG* mutant had only a slight effect on biofilm mass (Fig. 3f). Together, these findings suggested that the PDE activity of RpfG did not appear to be essential for its regulation of biofilm formation, further revealing that the repression of the DSF system in biofilm formation was independent of the PDE activity of RpfG in *L. enzymogenes*.

### WspR directly interacted with RpfG

**Fig. 4:**
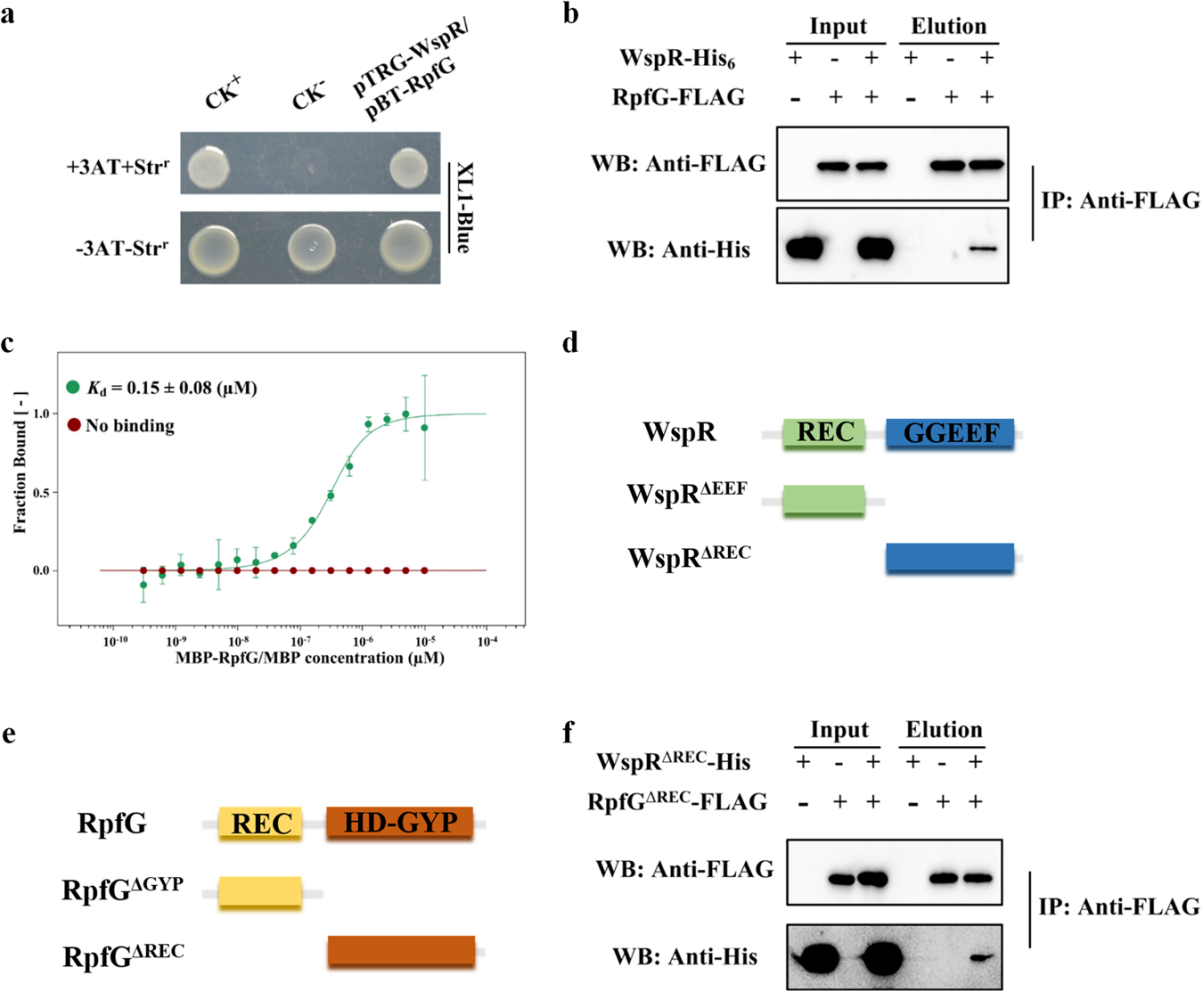
Physical interaction between WspR with RpfG. **a** E. coli-based B2H assay showing that WspR interacts with RpfG. CK^+^, positive control (pBT-GacS and pTRG-GacS) and CK^−^, negative control (empty vector of pBT and pTRG). WspR, pTRG-WspR. RpfG, pBT-RpfG. −3AT-Str^r^ and +3AT + Str^r^ represent strains grown on LB-based non-selective medium and minimal medium (M9)-based selective medium respectively. **b** Pull-down assay confirming the interaction of WspR-His with RpfG-FLAG. The IP assay was performed using anti-FLAG antibody. Western blotting was carried out using anti-FLAG and anti-His antibodies. **c** Microscale thermophoresis (MST) measurement of binding affinity: green trace, WspR-His and MBP-RpfG (*K*_d_ = 0.15 μM); red trace, WspR-His and MBP (no binding). **d** Schematic map of the full-length WspR and their truncations (WspR^ΔREC^ and WspR^ΔEEF^). **e** Schematic map of the full-length RpfG and their truncations (RpfG^ΔREC^ and RpfG^ΔGYP^). **f** Pull-down assay confirming the interactions between the GGDEF domains of WspR (WspR^ΔREC^) and the HD-GYP domain of RpfG (RpfG^ΔREC^). All blots derive from the same experiment and that they were processed in parallel.

Since both the Wsp and DSF system were involved in regulating biofilm formation, we were interested in understanding whether they could be interconnected. A previous study showed that RpfG regulates *Xcc* motility by binding to a number of GGDEF domain-containing proteins^47^. This knowledge promoted us to test whether RpfG could bind GGDEF-containing WspR. Bacterial two-hybrid (B2H) assay revealed that RpfG did bind to WspR (Fig. 4a). Pull-down assay involving WspR-His and RpfG-FLAG protein further confirmed this observation (Fig. 4b). To quantify WspR-RpfG binding affinity, we employed a microscale thermophoresis (MST) method, and our results showed that WspR-His bound to MBP-RpfG with moderately strong affinity (*K*_d_, 0.15 μM; Fig. 4c). A series of protein domain truncations further showed that the GGEEF domain of WspR interacted directly with the HD-GYP domain of RpfG (Fig. 4d-f). The above results indicated that WspR indeed interacted with RpfG. To further support the specific binding of WspR with RpfG, we expressed two additional proteins - LchD-His and MBP-LchP, which have been shown to be active DGC and PDE, respectively, in *L*. *enzymogenes*^41,45^. MST analysis showed that the intracellular portion of LchD (LchD^GGDEV^-His) did not interact with RpfG and WspR did not interact with MBP-LchP (Supplementary Fig. 7).

Because the DGC activity of WspR, but not the PDE activity of RpfG, is crucial for biofilm formation, we tested whether the enzymatic activity of WspR contributes to its binding to RpfG. We changed the GGDEF motif to GGAAF within the GGDEF domain to block the DGC activity of WspR. This step did not seem to affect the direct binding of the GGDEF domain of WspR to the HD-GYP domain of RpfG (Supplementary Fig. 8). Next, we tested whether c-di-GMP affects WspR-RpfG binding. By MST, we found that the addition of c-di-GMP in the physiological range (10 μM) exhibited only a slight effect on the WspR-RpfG interaction (*K*_d_, 0.32 μM; Supplementary Fig. 9). Together, these results suggested that WspR bound directly to RpfG and that c-di-GMP did not appear to disrupt the formation of the WspR-RpfG complex under the conditions tested.

### RpfG directly reduced DGC activity of unphosphorylated WspR

As previously mentioned, WspR regulates biofilm formation through its enzymatic activity, whereas RpfG does not appear to do so. Therefore, we wondered whether RpfG could act as an adaptor protein independent of its PDE activity to bind WspR and alter its enzymatic activity, thereby triggering WspR-dependent c-di-GMP signaling to regulate biofilm formation. To facilitate this investigation, we first examined RpfG^H190A^, an inactive RpfG variant^41^. As shown in Supplementary Fig. 10, native MBP-RpfG exhibited potent PDE activity to degrade c-di-GMP added to the test PDE buffer, whereas the RpfG^H190A^ variant exhibited significantly attenuated activity in the same buffer.

**Fig. 5:**
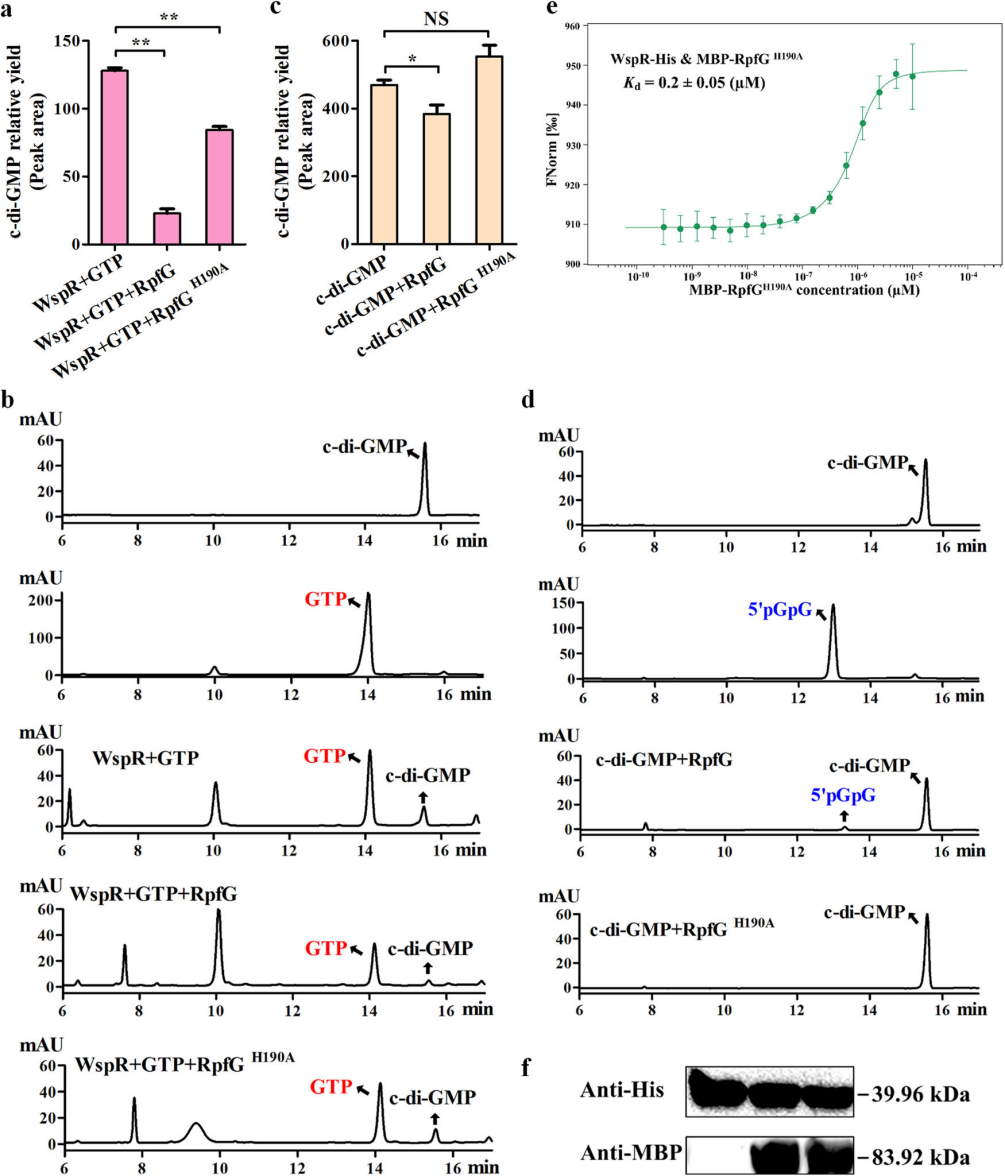
Interaction with RpfG impaired DGC activity of unphosphorylated WspR. **a** Decreased c-di-GMP synthesis by purified WspR-His in the presence of MBP-RpfG or MBP-RpfG^H190A^ using GTP as a substrate. The peak area of the HPLC chromatogram is expressed as the relative yield of c-di-GMP (y-axis). MBP-RpfG^H190A^ represents an enzymatically inactive variant of MBP-RpfG. **b** HPLC chromatogram corresponding to **a**. The c-di-GMP and GTP standard are represented in black and red, respectively. **c** The purified MBP-RpfG still exhibited slight enzymatic activity against c-di-GMP in vitro, but not MBP-RpfG^H190A^. **d** HPLC chromatogram corresponding to **c**. The 5′pGpG standard is represented in blue. **e** MST showing that WspR-His interacts with MBP-RpfG^H190A^ with moderate affinity (*K*_d_, 0.2 μM). **f** Western blotting showing that the reaction system in **a** did not affect protein stability. Anti-MBP and anti-His antibodies were used to detect WspR (39.96 kDa), RpfG, or RpfG^H190A^ (83.92 kDa). All blots derive from the same experiment and that they were processed in parallel. Statistical comparisons were performed using one-way ANOVA of GraphPad software (GraphPad, La Jolla, CA). In panels **a** and **c**, mean data ± SD from three experiments were shown, *P < 0.05; **P < 0.01. NS means not significant.

Next, we examined the relative amount of c-di-GMP synthesized by WspR by using GTP as a substrate in the presence or absence of RpfG or its RpfG^H190A^ variant. The results showed that the addition of native MBP-RpfG and MBP-RpfG^H190A^ reduced c-di-GMP production by 82% and 34% compared to the positive control (WspR-His) that efficiently synthesized c-di-GMP in the test buffer (Fig. 5a, b). Therefore, these findings suggested that RpfG may function as an adaptor protein to reduce the DGC activity of WspR. However, this result might also be due to the direct degradation of c-di-GMP by RpfG in the test DGC buffer. We could eliminate the latter possibility as we observed that the RpfG^H190A^ variant completely lost its ability to degrade the added c-di-GMP standard in the same DGC buffer without GTP (Fig. 5c, d). We thus speculated that RpfG most likely acted as an adaptor protein to reduce the DGC activity of WspR independent of its PDE activity. The following two evidences further supported this speculation. (i) The binding affinity of RpfG^H190A^ with WspR as determined by MST (*K*_d_, 0.2 μM; Fig. 5e) was similar to that of the RpfG-WspR complex (*K*_d_, 0.15 μM; Fig. 4c). (ii) The protein stability of MBP-RpfG and MBP-RpfG^H190A^ in DGC buffer was similar to that determined by Western blot (Fig. 5f). Therefore, our findings indicated that RpfG acted as an adaptor protein to reduce the DGC activity of unphosphorylated WspR.

### Phosphorylation of WspR not only impaired its binding affinity with RpfG, but also blocked the RpfG’s ability to act as an adaptor protein

**Fig. 6:**
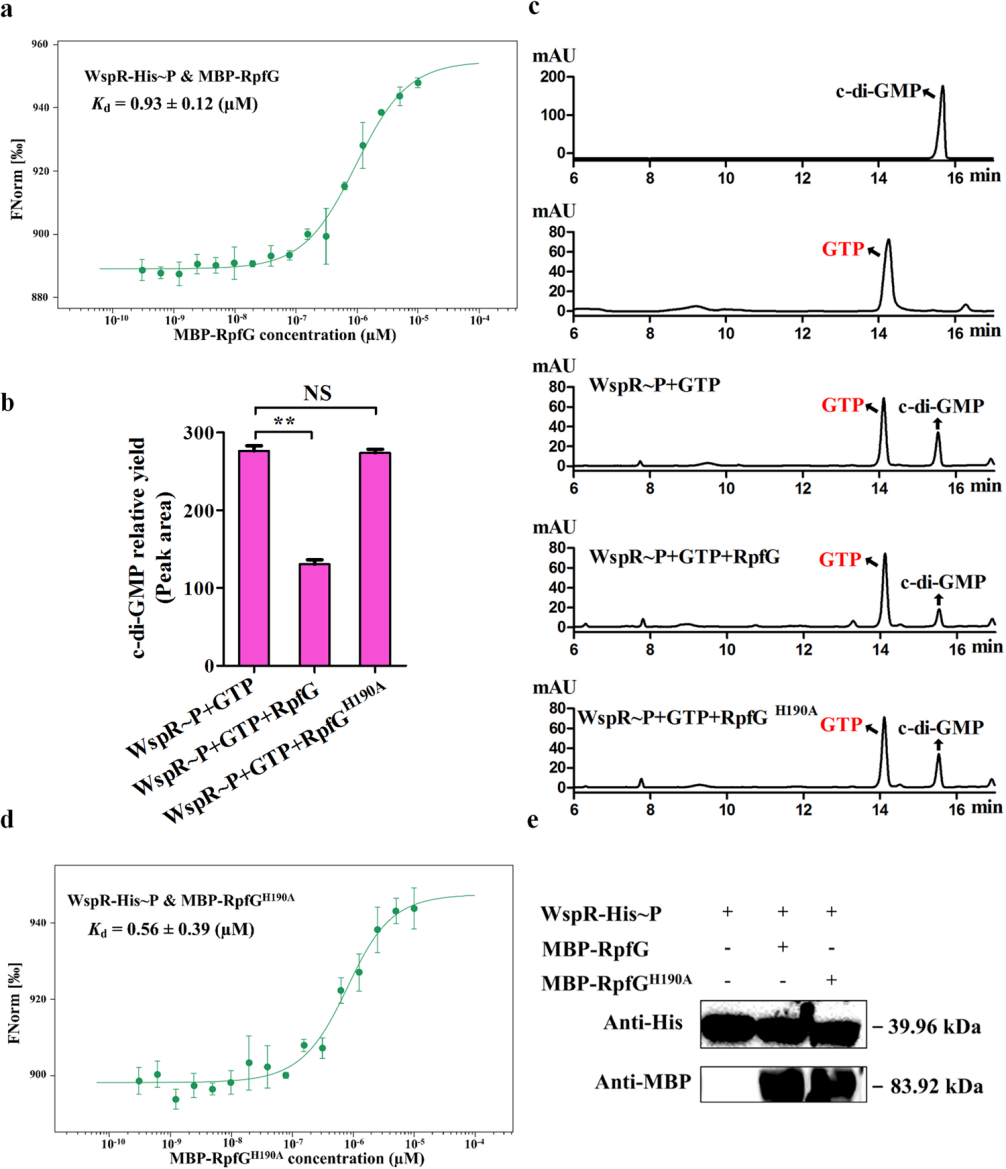
Phosphorylation of WspR decreases its binding affinity to RpfG and blocks the capacity of RpfG to function as an adaptor protein. **a** MST showing that phosphorylated WspR (WspR-His~P) interacts with MBP-RpfG with moderate affinity (*K*_d_, 0.93 μM). **b** Phosphorylation of WspR (WspR~P) reduces c-di-GMP synthesis in the presence of MBP-RpfG using GTP as a substrate but not MBP-RpfG^H190A^. The peak area of the HPLC chromatogram is expressed as the relative yield of c-di-GMP (y-axis). MBP-RpfG^H190A^ represents an enzymatically inactive variant of MBP-RpfG. **c** HPLC chromatogram corresponding to **b**. The c-di-GMP and GTP standard are represented in black and red, respectively. **d** MST showing that WspR-His~P interacts with MBP-RpfG^H190A^ with moderate affinity (*K*_d_, 0.56 μM). **e** Western blotting showing that the reaction system in **b** did not affect the stability of the tested protein. Anti-MBP and anti-His antibodies were used to detect WspR~P (39.96 kDa), RpfG, or RpfG^H190A^ (83.92 kDa). All blots derive from the same experiment and that they were processed in parallel. Statistical comparisons were performed using one-way ANOVA of GraphPad software (GraphPad, La Jolla, CA). In panels **b**, mean data ± SD from three experiments were shown, **P < 0.01. NS means not significant.

Our findings above clearly indicated that RpfG could act as an adaptor protein to bind and inhibit the DGC activity of WspR independently of its PDE activity; however, the signals or states that triggered the binding or dissociation of the WspR-RpfG complex remained elusive. To address this, we performed the following assays. First, we tested whether DSF addition affects the WspR-RpfG binding. By MST assay, we found that the addition of 10 μM DSF restored the biofilm phenotype of the *rpfF* mutant to wild type (Fig. 3d) and exhibited a slight increase in WspR-RpfG binding affinity (*K*_d_, 0.25 μM; Supplementary Fig. 11a) compared to the case without DSF supplement (*K*_d_, 0.15 μM). Second, we also prepared phosphorylated RpfG (RpfG~P) in vitro using acetyl phosphate according to the protocol described in a recent study in our laboratry^42^. The results of the MST assays showed that phosphorylation of RpfG in vitro only slightly increased the binding affinity to WspR (*K*_d_, 0.24 μM; Supplementary Fig. 11b). Third, we tested whether phosphorylation of WspR (WspR~P) affects WspR-RpfG binding. MST results showed that although WspR~P also bound to RpfG, their binding affinity (*K*_d_, 0.93 μM; Fig. 6a) decreased by approximately 6.2 folds compared with that of unphosphorylated WspR to RpfG (*K*_d_, 0.15 μM). Together, these results uncovered that the WspR-RpfG complex did not appear to be disassembled upon phosphorylation of WspR or RpfG, although phosphorylation of WspR significantly decreased its binding affinity to RpfG.

Since we found that the DGC activity of WspR could be attenuated by binding to RpfG independently of its PDE activity, we tested whether this also happened when WpsR is phosphorylated. For this purpose, we examined the relative amounts of c-di-GMP synthesized by phosphorylated WspR using GTP as a substrate in the presence or absence of RpfG or its PDE-inactive RpfG^H190A^ variant. The results showed that the addition of native MBP-RpfG reduced c-di-GMP production by 52% compared to the positive control (WspR~P; Fig. 6b, c). Under similar testing conditions, addition of the same concentration of MBP-RpfG^H190A^ variant never inhibited WspR~P -derived c-di-GMP production (Fig. 6b, c). In this case, RpfG^H190A^ also efficiently bound to WspR~P (*K*_d_, 0.56 μM; Fig. 6d) and MBP-RpfG and MBP-RpfG^H190A^ had similar protein stability under the test conditions (Fig. 6e). Taken together, our results revealed that phosphorylation of WspR caused a dual effect; it not only weakened its binding affinity to RpfG, but also blocked the ability of RpfG to act as an adaptor protein to inhibit the DGC activity of WspR~P.

## Discussion

Plant-beneficial bacteria live in diverse natural ecological niches. The formation of biofilms on host plants or pathogens is a prerequisite for them to protect plants or kill pathogens^48^. For example, rhizosphere bacteria (*Stenotrophomonas rhizophila* DSM14405^T^) promote plant growth by regulating the biofilm formation in plant rhizosphere in harsh natural environments^25^. However, the mechanisms by which plant-beneficial bacteria fine-tune biofilm formation are only partly uncovered. Here, we present a novel mechanism found in the soil bacterium *L. enzymogene*s. We found that the Wsp chemosensory system integrated DSF quorum sensing to regulate biofilm formation through c-di-GMP signaling involving the WspR-RpfG complex. Although the Wsp and DSF systems are known to control biofilm formation in pathogenic *P. aeruginosa* and *X. campestris*^9–11^, our findings pointed out three distinct features compared to earlier observations: (i) The function of the DSF system in pathogenic bacteria depended on the PDE activity of RpfG, whereas we found that DSF regulated biofilm formation independently of the PDE activity of RpfG in the plant-beneficial bacterium *L. enzymogenes*. (ii) The crosstalk between the Wsp and DSF systems in pathogenic bacteria has not been reported, whereas we uncovered that both systems can establish crosstalk through the formation of WspR-RpfG complex in *L. enzymogenes*. (iii) WspR-RpfG interaction triggered an unusual c-di-GMP signaling effect, in which the active PDE RpfG could act as an adaptor protein to alter the DGC activity of unphosphorylated WspR independent of its enzymatic activity. (iv) Phosphorylation of WspR had a dual effect; it not only impaired the binding affinity to RpfG, but also blocked the ability of RpfG to act as an adaptor protein to inhibit the DGC activity of phosphorylated WspR. In general, active DGC-PDE formation of protein complexes is thought to maintain local c-di-GMP dynamics, both of which are dependent on their DGC and PDE activities^15,49^.

**Fig. 7:**
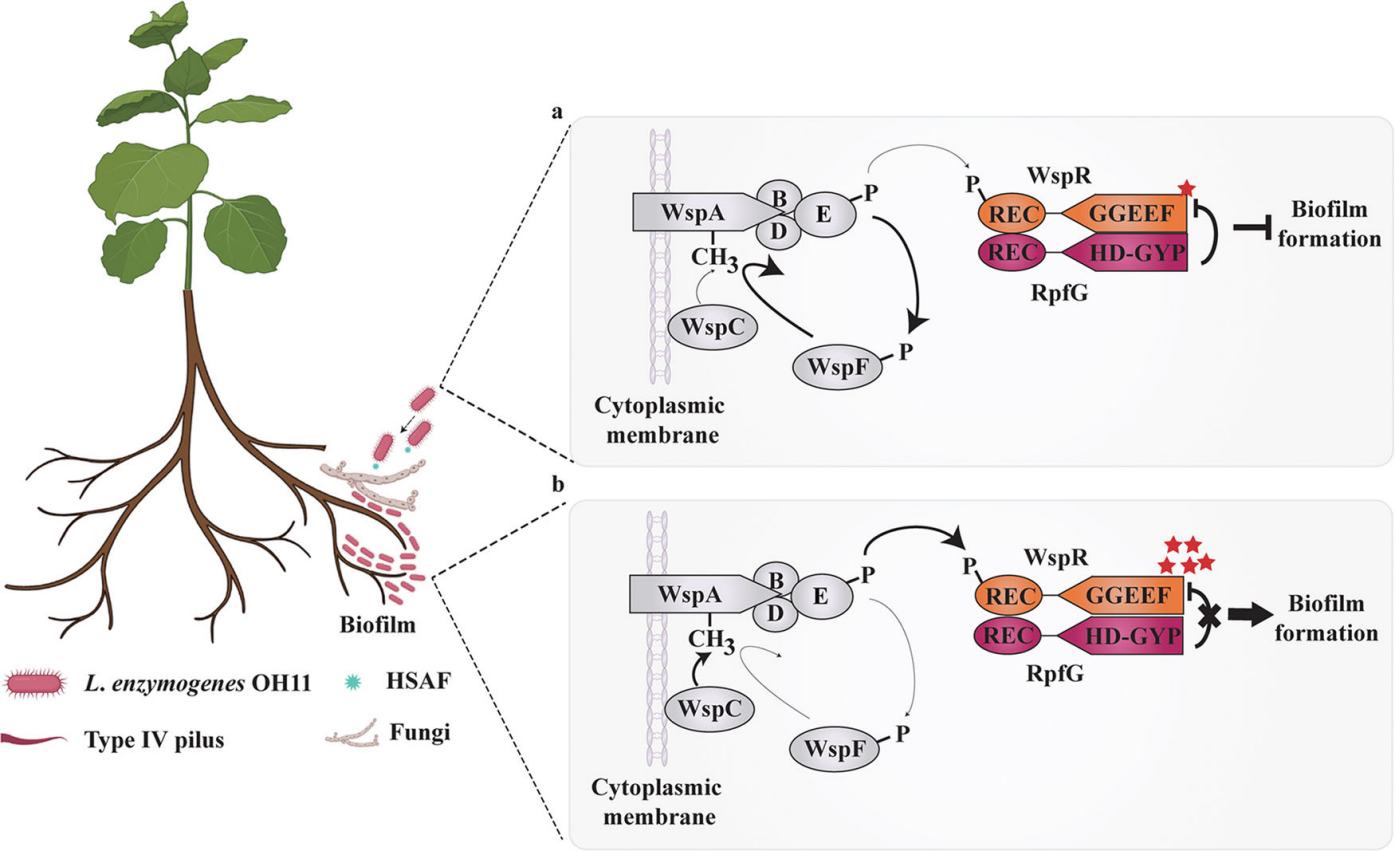
The proposed model showing how the Wsp system controls c-di-GMP-dependent biofilm formation by integrating the DSF system in *L. enzymogenes*. *Lysobacter enzymogenes* OH11 is a member of the soil microbiome and a natural predator of fungal pathogens that infect crop roots. As previously described, in the presence of nearby fungal pathogens (dark lightening symbol), this bacterium most likely forms planktonic cells to facilitate its movement towards fungi via T4P and inhibit fungal growth by secreting the antifungal antibiotic HSAF^42^,. **a** At this point, WspA, an MCP-like protein, blocks auto-phosphorylation of histidine kinase WspE by inhibiting WspC activity (thin dark arrows) and/or promoting the WspF activity (thick dark arrows), resulting in un-phosphorylated WspR that displays decreased DGC activity. Meanwhile, the active PDE RpfG of the DSF system can act as an adaptor protein to bind to WspR independent of its PDE activity and further inhibit its DGC activity, resulting in attenuated intracellular c-di-GMP levels, thereby inhibiting biofilm formation. **b** When *L. enzymogenes* cells attach to fungal hyphae in the root microenvironment, WspA appears to be in a methylation state by enhancing the WspC activity (a thick dark arrow) and/or inhibiting the WspF activity (a thin dark arrow) to stimulate the auto-phosphorylation of WspE, thereby producing phosphorylated WspR (WspR~P; thicker arrow). In this case, WspR~P not only impairs its binding affinity to RpfG, but also blocks the ability of RpfG to act as an adaptor protein to inhibit the DGC activity of WspR~P, resulting in elevated intracellular c-di-GMP concentration that promotes biofilm formation via bacterial colonization. This figure was created with BioRender.com.

As mentioned earlier, *L. enzymogenes* is a natural predator of fungi^37^. During predation, it approaches nearby fungi via T4P (type IV pilus)-driven twitching motility, allowing bacteria to form planktonic cells^40,50^. In this case, we speculate that WspA is demethylated by the methylesterase WspF to inhibit the autophosphorylation of WspE, thereby preventing the transfer of the phosphate group to WspR. Unphosphorylated WspR exhibits lower c-di-GMP synthesis activity^42^. Binding of RpfG further inhibits the enzymatic activity of unphosphorylated WspR, thereby reducing intracellular c-di-GMP levels, resulting in reduced biofilm formation (Fig. 7a). Consistent with this case, in our previous study, we have found that unphosphorylated WspR also binds to CdgL, which interacts further with LysR, most likely to form a WspR-CdgL-LysR ternary complex to enhance HSAF production, thus helping this non-flagellated bacterium to move towards nearby fungi via T4P and suppresses their growth, which is promoted by low level of c-di-GMP^40,45^. When *L. enzymogenes* cells attach to the fungal hyphae, it might efficiently secrete lyases to hydrolyze the fungal hyphae and switch the lifestyle to a sessile state by forming a biofilm^40^. In this case, WspC could methylate WspA to activate WspE for autophosphorylation and transfer the phosphate group to WspR. Phosphorylation of WspR not only impairs its binding affinity to RpfG, but also blocks the ability of RpfG to act as an adaptor protein to inhibit the DGC activity of phosphorylated WspR, resulting in elevated intracellular c-di-GMP concentration and promotion of biofilm formation, thereby helping *L*. *enzymogenes* colonize solid surfaces or resisting adverse external environments (Fig. 7b).

It is noteworthy that our findings, along with earlier reports, uncovered that the Wsp system regulated biofilm formation through distinct c-di-GMP receptors in the flagellated *P*. *aeruginosa* and the non-flagellated *L. enzymogenes*^17,42^. In *P*. *aeruginosa*, when WspA senses solid surface or fatty acid signals, it activates WspE to transfer phosphate group to WspR^16^. Phosphorylation of WspR increases intracellular c-di-GMP levels, thereby affecting downstream signaling output through the binding of c-di-GMP to the transcription factor FleQ. FleQ interacts with c-di-GMP to not only repress the expression of flagellar genes, but also promote the transcription of numerous genes involved in extracellular polysaccharide production and biofilm formation, thereby transforming cells from a planktonic state to a sessile lifestyle^51^. *L. enzymogenes* did not seem to employ this FleQ-dependent c-di-GMP signaling to regulate biofilm formation, because this non-flagellated bacterium has evolutionally lost FleQ homology^42^. Alternatively, we previously found that CdgL is a c-di-GMP receptor located downstream of the Wsp system in *L. enzymogenes*^43^. Direct binding of CdgL to WspR led to c-di-GMP-dependent inhibition of antifungal HSAF production^42,43^. However, we found that CdgL did not appear to be involved in the regulation of biofilm formation, as *cdgL* mutants formed similar biofilm biomass to wild-type OH11 (Supplementary Fig. 12). This suggested that the Wsp system could employ a yet-to-be identified c-di-GMP receptor to control biofilm formation in *L. enzymogenes*, suggesting that the signaling complexity of c-di-GMP is responsible for distinct Wsp functional outputs (HSAF or biofilm). Although we have not yet identified such a c-di-GMP receptor, the transcription factor Clp is considered to be a potential candidate based on the following considerations: (i) Clp binds strongly to c-di-GMP, but not to WspR^42,45^, which is similar to the case of FleQ^52^. (ii) The *clp* mutant produced more biofilms than wild type (Supplementary Fig. 12), similar to the *wspF* or *rpfG* mutants. (iii) Clp is shown to be downstream of DSF signaling, but fails to directly interact with RpfG^11^.

Notably, the active PDE RpfG could regulate biofilm formation independently of its enzymatic activity in *L. enzymogenes*, in contrast to the situation in the phytopathogen *X. campestris* and the plant-beneficial bacterium *S. rhizophila* DSM14405^T^ ^11,25^. However, although we did not have detailed information to explain this difference, a recent study showed that RpfG regulates HSAF biosynthesis also independently of its PDE activity^41^. We proposed that establishing direct interactions with other proteins through RpfG seemed to be one of the reasons why its mode of action was independent of its PDE activity. Indeed, RpfG is known to interact with three hybrid two-component system (HyTCS) proteins, HtsH1, HtsH2, and HtsH3, to control HSAF production^41^. Here, we reported that RpfG could act as an adaptor protein to regulate the DGC activity of WspR.

Bacteria typically have large amounts of DGCs and PDEs associated with c-di-GMP signaling^49^. Numerous studies have demonstrated that physical interactions between certain DGCs and PDEs can maintain local c-di-GMP signaling, thereby inducing specific c-di-GMP-dependent functional outputs. Typical examples include, but not limit to, complexes formed by DGC LchD and PDE LchP from *L*. *enzymogenes*, DGC YdaM and PDE YciR, DGC DgcC and PDE PdeK, and DGC DosC and PDE DosP from E. coli^46,53–56^. In these reported cases, it is generally believed that the local c-di-GMP levels maintained by these DGC-PDE complexes are all dependent on their enzymatic activity^15,49^. However, we provided evidences that RpfG, an active PDE, seemed to maintain local c-di-GMP with WspR independent of its enzymatic activity. Alternatively, it appeared to act in an unusual manner as an adaptor protein to bind and modify WspR enzymatic activity. Since RpfG is known to directly interact with various c-di-GMP-associated, GGDEF-containing proteins in pathogenic *X. campestris*^11^, our findings also revealed the unique biochemical properties of these protein-protein interactions involving RpfG.

## Methods

### Bacterial strains, plasmids and growth conditions

We listed the strains and plasmids used in this study in Supplementary Table 1. E. coli DH5α for plasmid construction was aerobically grown in Lysogenic-Broth (LB) medium (10 g/L tryptone, 10 g/L NaCl, 5 g/L yeast extract [pH 7.0]) at 37 °C with appropriate antibiotics (30 μg/ml gentamicin, Gm). *L. enzymogenes* OH11 and its derivative strains were cultivated in LB medium at 28 °C with appropriate antibiotics (100 μg/mL kanamycin, Km for mutant construction, and 150 μg/mL Gm for plasmid maintenance).

### Genetic methods

In-frame deletions in *L. enzymogenes* OH11 were generated using an established method^57^. In short, the upstream and downstream regions of the target gene were PCR-amplified using the corresponding primers (Supplementary Table 2) and cloned into the suicide vector pEX18Gm (Supplementary Table 1). Afterwards, the recombinant vectors were transformed into the wild-type strain by electroporation. Subsequently, single-crossover colonies were selected on LB plates supplemented with 100 μg/mL Km and 150 μg/mL Gm. Positive transformants were cultured in LB medium without any antibiotics at 28 °C for 6 h, then plated on LB agar including 10% (w/v) sucrose and 100 μg/mL Km to select for double-crossover colonies. Finally, the in-frame deletions were confirmed by PCR using the corresponding primers (Supplementary Table 2).

To construct the chromosomal complementation or point mutation in *L. enzymogenes* OH11, the target fragments, including the coding and upstream and downstream regions of each gene, with or without point mutations, were amplified by PCR using the corresponding primers (Supplementary Table 2). The purified PCR fragments were cloned into pEX18Gm to create recombinant vectors (Supplementary Table 1), and then transformed into wild type or mutants by electroporation. Screening and PCR verification of positive transformants were consistent with the case described above.

To construct the overexpression strains, the target fragments, including coding regions and their predicted promoters, were PCR-amplified using the corresponding primers^58^ (Supplementary Table 2). The purified DNA was cloned into the broad-host vector pBBR1-MCS5 (Supplementary Table 1). To obtain an overexpression strain, the recombinant vector was transformed into a wild-type strain by electroporation, and the resulting over-expression strain was confirmed by PCR.

### Biofilm biomass assay

Biofilm biomass assays were performed as described by Wang et al.^59^. Briefly, *L. enzymogenes* OH11 and its derivative strains were cultured in LB medium to a final concentration of OD_600_ = 1.0. After that, 4 mL of bacterial suspension were transferred to sterilized culture tubes, which were incubated in a constant temperature incubator at 28 °C for 24 h without shaking. The medium was removed and the tubes washed 3 times in pure water. Biofilm biomass in the tubes was visualized by adding 4 mL 0.1% CV followed by 3 washes in pure water. CV-stained biofilms in culture tubes were washed with eluent (methyl alcohol: acetic acid: water = 4:1:5, v/v/v). We then added 200 μL of the eluent to a 96-well plate and measured absorbance (OD_575_) using an Agilent 8453 UV–visible spectrophotometer (Agilent Technologies, Inc., Santa Clara, CA, USA).

For confocal-based biofilm assays, we used the method described by Du, 2016^60^. Briefly, the GFP-expressing plasmid pBBR1-MCS5 was transformed into *L. enzymogenes* OH11 and its derivative strains. GFP-labelled strains were cultured overnight in LB medium and adjusted to OD_600_ = 1.0, diluted to 1% in fresh LB medium, and 300 μL of the cultures were added to chamber-covered slides (Nu155411, Lab-Tek, NUNC, Naperville, IL, USA). Chambers were kept in a constant temperature incubator at 28 °C for 24 h without shaking. 3D images of biofilms were visualized by a laser scanning confocal microscopy (Leica Microsystems Inc., Buffalo Grove, IL, USA) with a 20× objective. The excitation wavelength is 488 nm, and the green fluorescence absorption is 500–545 nm. LAS_X_Small_2.0.0_14332 software was used for analyzing 3D images.

### Bacterial two-hybrid (B2H) assays

The BacterioMatch II Two-Hybrid system (Agilent Technologies, CA, USA) was used to rapidly detect possible interactions between proteins. The B2H assay was carried out according to a procedure used in laboratory^45^. In short, target genes containing coding regions were PCR-cloned into pBT and pTRG vectors, respectively, and these were transformed into E. coli XL1-Blue MRF ´ Kan. The vectors pBT-GacS and pTRG-GacS were used as positive controls^45^ and empty pTRG and pBT plasmids as negative controls. All co-transformed strains were spotted onto selective agar plates (selective agar, denoted as +3AT + Str^r^) and cultured at 28°C for 2 to 3 days. Strains with pBT-RpfG and pTRG-WspR would be expected to grow well on the selective agar plates if there is a direct physical interaction between WspR and RpfG. Selective agar consisted of minimal medium (M9) supplemented with 30 μg/mL kanamycin, 34 μg/mL chloramphenicol, 12.5 μg/mL tetracycline, 5 mM 3-AT, and 8 μg/mL Str^45^. LB agar is nonselective (denoted as -3AT-Str^r^) and comprises 12.5 μg/mL tetracycline, 34 μg/mL chloramphenicol, and 30 μg/mL kanamycin^45^. The purpose of LB agar is to confirm that all recombinant vectors were successfully transformed into E. coli XL1-Blue MRF ´ Kan.

### Protein expression and purification

Protein expression and purification were carried out according to an established method^42^. Briefly, the coding regions of WspR and its derivatives were amplified by PCR using the corresponding primers (Supplementary Table 2) and cloned into the vector pET30a to generate fusion proteins with His-tag. Afterwards, the recombinant vectors were transformed into E. coli BL21 (DE3), overexpressed, and purified with pre-equilibrated Ni^2+^ resin (GE Healthcare, Shanghai, China). BCA protein assay kit (Sangon Biotech, Shanghai, China) and SDS-PAGE were used to determine protein concentration or purity, respectively. Expression and purification of MBP-RpfG was performed as described by Li et al.^41^. The coding regions of *rpfG* gene was amplified and inserted into pMAl-p2x to produce the plasmids pMAl-*rpfG*. RpfG and RpfG site-directed mutants with a vector-encoded maltose binding protein were expressed in E. coli BL21 (DE3) and purified with Dextrin Sepharose High Performance (Qiagen, Chatsworth, CA, USA) using an affinity column (Qiagen).

### DGC and PDE activity assays in vitro

The PDE activity assay was performed^61^ using 2 μM of MBP-RpfG or MBP-RpfG^H190A^ in 10 mM MgCl_2_, 60 mM Tris–HCl (pH 7.6), 10 mM MnCl_2_ and 50 mM NaCl. The degradation started with the addition of 100 μM c-di-GMP.

DGC activity assay was performed^62^ using 10 μM of WspR-His in 10 mM MgCl_2_, 75 mM Tris–HCl (pH 8.0), 25 mM KCl and 250 mM NaCl. The synthesis reaction was started by the addition of 150 μM GTP. All reaction samples were incubated at 30 °C for 1 or 2 h and then boiled for 10 min to stop the reaction. The mixtures were filtered through a 0.22 μM pore size cellulose-acetate filter. 20 μl of each mixture was loaded onto a reverse-phase C18 column and separated by HPLC. Two mobile phases, 100 mM KH_2_PO_4_ + 4 mM tetrabutylammonium sulfate (A) and 75% A + 25% methanol (B), were used for the separation procedure.

### Pull-down assays

All possible interactions between proteins in vitro were examined using pull-down assay^45^. *wspR* was cloned into pET30a with a C-terminal His-tag, while *rpfG* was cloned into pET30a fused to a C-terminal FLAG-tag. Both WspR and RpfG were expressed in E. coli BL21 (DE3) and induced by 0.8 mM IPTG. 1 mL of bacteria lysate containing WspR-His or RpfG-FLAG was then incubated with 10 μL of anti-FLAG magnetic beads (Bimake, Shanghai, China). After overnight incubation at 4 °C, the beads were washed 3 times for 5 min each with 1 ml of 10 mM PBS buffer (pH, 7.5) containing 1% Triton X-100. Proteins bound to the beads were eluted with 45 μl elution buffer (0.2 M glycine HCl, pH 3.0), followed by the addition of 5 μl neutralization buffer (1 M Tris, pH 10). The eluted samples were boiled in 4×SDS loading buffer for 8 min. These samples were loaded into SDS-PAGE for Western blotting, and proteins were detected using anti-FLAG (No. M20008S, Abmart) and anti-His-tag (No. M30111L, Abmart) from Shanghai, China. Uncropped and unprocessed scans of gels & blots were provided in Supplementary Fig. 13.

### Microscale thermophoresis (MST) assays

Dissociation constants of protein-protein interaction were detected by Microscale Thermophoresis (MST) using Monolith NT.115 (NanoTemper Technologies, Germany)^44,45^. For the WspR-RpfG/ RpfG^H190A^ binding assay, purified WspR-His was labeled with the fluorescent dye RED-Tris-NTA (NanoTemper Technologies, Germany). A constant concentration (100 nM) of labeled WspR was titrated against increasing concentrations of MBP-RpfG or MBP-RpfG^H190A^ in standard MST buffer (50 mM Tris, pH 7.5, 150 mM NaCl, 10 mM MgCl_2_, and 0.05% Tween 20). The mixtures were loaded into the MST device using a high-precision capillary (Monolith NT.115 MO-K022, Germany) at 25 °C using medium MST power and 60% LED power. FNorm was plotted on a linear y-axis in per mil (‰) on the log_10_ x-axis relative to the total concentration of titration partner^63^. The data were analyzed using Nanotemper Analysis software v.1.2.101 (NanoTemper Technologies, Germany).

### C-di-GMP extraction and quantification

Cultures were grown in LB medium at 28°C until OD_600_, 1.5. Cells from 2 ml culture were harvested for protein quantification by the BCA assay (TransGen, China). Cells from 8 mL of culture were used for c-di-GMP extraction utilizing 0.6 M HClO_4_ and 2.5 M K_2_CO_3_^45^. Samples were assayed by liquid chromatography-tandem mass spectrometry (LC-MS/MS) analysis on an AB SCIEX QTRAP 6500 LC-MS/MS system^41^.

### Statistical analysis

Statistical analyses were performed using GraphPad Prism (version 8.0.0). In all assays, mean data from three experiments were shown with ± SD (standard deviation). Statistical significance was determined using one-way ANOVA. *p* values were reported using the following symbolic representation: NS (No significance) *p* > 0.05, *p < 0.05, **p < 0.01.

### Reporting Summary

Further information on research design is available in the Nature Research Reporting Summary linked to this article.

## Supplementary information

Supporting information

Reporting summary

## Data Availability

The sequence data from the present study have been submitted to the NCBI GenBank under the following accession numbers: MT157314 (Le4555; WspA), MT157315 (Le4557; WspB), MT157316 (Le4558; WspC), MT157317 (Le4559; WspD), MT157318 (Le4560; WspE), MT157319 (Le4561; WpsF), Le4562 (MG387209; WspR), MG387215.1 (Le4727; RpfG) and MG387193.1 (Le2762; LchP). The data that support the findings of this study are available in the main article, Supplementary information files or from the corresponding author upon reasonable request. The source data of dots and gels are provided in Supplementary Fig. 13.

## References

[1] Saunders SH (2020). Extracellular DNA promotes efficient extracellular electron transfer by pyocyanin in *Pseudomonas aeruginosa* biofilms. Cell.

[2] Flemming HC (2016). Biofilms: an emergent form of bacterial life. Nat. Rev. Microbiol..

[3] Flemming HC, Wingender J (2010). The biofilm matrix. Nat. Rev. Microbiol..

[4] Vestby LK, Grønseth T, Simm R, Nesse LL (2020). Bacterial biofilm and its role in the pathogenesis of disease. Antibiotics (Basel).

[5] Guzmán-Soto I (2021). Mimicking biofilm formation and development: recent progress in in vitro and in vivo biofilm models. iScience.

[6] Rather MA, Gupta K, Mandal M (2021). Microbial biofilm: formation, architecture, antibiotic resistance, and control strategies. Braz. J. Microbiol.

[7] Valentini M, Filloux A (2019). Multiple roles of c-di-GMP signaling in bacterial pathogenesis. Annu. Rev. Microbiol..

[8] Randall TE (2022). Sensory perception in bacterial cyclic diguanylate signal transduction. J. Bacteriol..

[9] Valentini M, Filloux A (2016). Biofilms and cyclic di-GMP (c-di-GMP) signaling: lessons from *Pseudomonas aeruginosa* and other bacteria. J. Biol. Chem..

[10] Simm R (2004). GGDEF and EAL domains inversely regulate cyclic di-GMP levels and transition from sessility to motility. Mol. Microbiol.

[11] Pfeilmeier S, Caly DL, Malone JG (2016). Bacterial pathogenesis of plants: future challenges from a microbial perspective: Challenges in Bacterial Molecular Plant Pathology. Mol. Plant. Pathol..

[12] Römling U, Galperin MY, Gomelsky M (2013). Cyclic di-GMP: the first 25 years of a universal bacterial second messenger. Microbiol. Mol. Biol. Rev..

[13] Jenal U, Reinders A, Lori C (2017). Cyclic di-GMP: second messenger extraordinaire. Nat. Rev. Microbiol..

[14] Liu C (2022). cAMP and c-di-GMP synergistically support biofilm maintenance through the direct interaction of their effectors. Nat. Commun..

[15] Hengge R (2021). High-specificity local and global c-di-GMP signaling. Trends Microbiol..

[16] Matilla MA, Martín-Mora D, Gavira JA, Krell T (2021). *Pseudomonas aeruginosa* as a model to study chemosensory pathway signaling. Microbiol. Mol. Biol. Rev..

[17] Hickman JW, Tifrea DF, Harwood CS (2005). A chemosensory system that regulates biofilm formation through modulation of cyclic diguanylate levels. Proc. Natl Acad. Sci. USA.

[18] Huangyutitham V, Guvener ZT, Harwood CS (2013). Subcellular clustering of the phosphorylated WspR response regulator protein stimulates its diguanylate cyclase activity. mBio.

[19] Huang Z, Pan X, Xu N, Guo M (2019). Bacterial chemotaxis coupling protein: structure, function and diversity. Microbiol. Res..

[20] Xu A (2022). Mutations in surface-sensing receptor WspA lock the Wsp signal transduction system into a constitutively active state. Environ. Microbiol..

[21] Porter SL, Wadhams GH, Armitage JP (2011). Signal processing in complex chemotaxis pathways. Nat. Rev. Microbiol..

[22] He K, Bauer CE (2014). Chemosensory signaling systems that control bacterial survival. Trends Microbiol..

[23] O’Neal L (2022). The Wsp system of *Pseudomonas aeruginosa* links surface sensing and cell envelope stress. Proc. Natl Acad. Sci. USA.

[24] Ryan RP, Dow JM (2011). Communication with a growing family: diffusible signal factor (DSF) signaling in bacteria. Trends Microbiol..

[25] Liu Y (2022). Diffusible signal factor enhances the saline-alkaline resistance and rhizosphere colonization of *Stenotrophomonas rhizophila* by coordinating optimal metabolism. Sci. Total. Environ..

[26] Deng Y, Wu J, Eberl L, Zhang LH (2010). Structural and functional characterization of diffusible signal factor family quorum-sensing signals produced by members of the *Burkholderia cepacia* complex. Appl. Environ. Microbiol..

[27] Papenfort K, Bassler BL (2016). Quorum sensing signal-response systems in Gram-negative bacteria. Nat. Rev. Microbiol..

[28] Singh P, Verma RK, Chatterjee S (2022). The diffusible signal factor synthase, RpfF, in *Xanthomonas oryzae* pv. *oryzae* is required for the maintenance of membrane integrity and virulence. Mol. Plant. Pathol..

[29] Dow JM (2017). Diffusible signal factor-dependent quorum sensing in pathogenic bacteria and its exploitation for disease control. J. Appl. Microbiol.

[30] He YW (2006). Dual signaling functions of the hybrid sensor kinase RpfC of *Xanthomonas campestris* involve either phosphorelay or receiver domain-protein interaction. J. Biol. Chem..

[31] Zhou L, Zhang LH, Cámara M, He YW (2017). The DSF family of quorum sensing signals: diversity, biosynthesis, and turnover. Trends Microbiol..

[32] Ryan RP (2015). The DSF family of cell-cell signals: an expanding class of bacterial virulence regulators. PLoS. Pathog..

[33] He YW, Zhang LH (2008). Quorum sensing and virulence regulation in *Xanthomonas campestris*. Fems. Microbiol. Rev..

[34] Solano C, Echeverz M, Lasa I (2014). Biofilm dispersion and quorum sensing. Curr. Opin. Microbiol..

[35] Ryan RP (2006). Cell-cell signaling in Xanthomonas campestris involves an HD-GYP domain protein that functions in cyclic di-GMP turnover. Natl Acad. Sci. USA.

[36] Ren X (2020). Knockout of diguanylate cyclase genes in *Lysobacter enzymogenes* to improve production of antifungal factor and increase its application in seed coating. Curr. Microbiol..

[37] Lin L (2021). Antifungal weapons of *Lysobacter*, a mighty biocontrol agent. Environ. Microbiol..

[38] Xu K (2021). Coordinated control of the type IV pili and c-di-GMP-dependent antifungal antibiotic production in *Lysobacter* by the response regulator PilR. Mol. Plant. Pathol..

[39] Yang M (2020). An intrinsic mechanism for coordinated production of the contact-dependent and contact-independent weapon systems in a soil bacterium. PLoS. Pathog..

[40] Zhao Y (2017). Transcriptional and antagonistic responses of biocontrol strain *Lysobacter enzymogenes* OH11 to the plant pathogenic oomycete *Pythium aphanidermatum*. Front. Microbiol..

[41] Li K (2021). The predatory soil bacterium *Lysobacter* reprograms quorum sensing system to regulate antifungal antibiotic production in a cyclic-di-GMP-independent manner. Commun. Biol..

[42] Xu K (2021). A non-flagellated, predatory soil bacterium reprograms a chemosensory system to control antifungal antibiotic production via cyclic di-GMP signalling. Environ. Microbiol..

[43] Han S (2020). A YajQ-LysR-like, cyclic di-GMP-dependent system regulating biosynthesis of an antifungal antibiotic in a crop-protecting bacterium, *Lysobacter enzymogenes*. Mol. Plant. Pathol..

[44] Su Z (2017). 4-Hydroxybenzoic acid is a diffusible factor that connects metabolic shikimate pathway to the biosynthesis of a unique antifungal metabolite in *Lysobacter enzymogenes*. Mol. Microbiol..

[45] Xu G (2018). Signaling specificity in the c-di-GMP-dependent network regulating antibiotic synthesis in *Lysobacter*. Nucleic Acids Res.

[46] Xu G, Zhou L, Qian G, Liu F (2022). Diguanylate cyclase and phosphodiesterase interact to maintain the specificity of cyclic di-GMP signaling in the regulation of antibiotic synthesis in *Lysobacter enzymogenes*. Appl. Environ. Microbiol..

[47] Ryan RP (2010). Cell-cell signal-dependent dynamic interactions between HD-GYP and GGDEF domain proteins mediate virulence in *Xanthomonas campestris*. Proc. Natl Acad. Sci. USA.

[48] Li Q (2018). FtsEX-CwlO regulates biofilm formation by a plant-beneficial rhizobacterium *Bacillus velezensis* SQR9. Res. Microbiol..

[49] Sarenko O (2017). More than enzymes that make or break cyclic di-GMP-local signaling in the interactome of GGDEF/EAL domain proteins of *Escherichia coli*. mBio.

[50] Xu K (2021). Clp is a “busy” transcription factor in the bacterial warrior, Lysobacter enzymogenes. Comput. Struct. Biotechnol. J..

[51] Baraquet C, Harwood CS (2016). FleQ DNA binding consensus sequence revealed by studies of FleQ-dependent regulation of biofilm gene expression in *Pseudomonas aeruginosa*. J. Bacteriol..

[52] Baraquet C, Murakami K, Parsek MR, Harwood CS (2012). The FleQ protein from *Pseudomonas aeruginosa* functions as both a repressor and an activator to control gene expression from the *pel* operon promoter in response to c-di-GMP. Nucleic Acids Res..

[53] Lindenberg, S. et al. The EAL domain protein YciR acts as a trigger enzyme in a c-di-GMP signalling cascade in. E. coli *biofilm control. Embo. j.* **32**, 2001–2014 (2013). 10.1038/emboj.2013.120 PMC3715855 23708798

[54] Dahlstrom KM (2015). Contribution of physical interactions to signaling specificity between a diguanylate cyclase and its effector. mBio.

[55] Richter AM (2020). Local c-di-GMP signaling in the control of synthesis of the E. coli biofilm exopolysaccharide pEtN-cellulose. J. Mol. Biol..

[56] Tuckerman JR (2009). An oxygen-sensing diguanylate cyclase and phosphodiesterase couple for c-di-GMP control. Biochemistry.

[57] Qian G (2012). Selection of available suicide vectors for gene mutagenesis using *chiA* (a chitinase encoding gene) as a new reporter and primary functional analysis of *chiA* in *Lysobacter enzymogenes* strain OH11. World. J. Microbiol. Biotechnol..

[58] Qian G (2014). Roles of a solo LuxR in the biological control agent *Lysobacter enzymogenes* strain OH11. Phytopathology.

[59] Wang B (2018). Dissecting the virulence-related functionality and cellular transcription mechanism of a conserved hypothetical protein in *Xanthomonas oryzae* pv. *oryzae*. Mol. Plant. Pathol..

[60] Du H (2016). Identification and characterization of an *Aeromonas hydrophila* oligopeptidase gene *pepF* negatively related to biofilm formation. Front. Microbiol..

[61] Schmidt AJ, Ryjenkov DA, Gomelsky M (2005). The ubiquitous protein domain EAL is a cyclic diguanylate-specific phosphodiesterase: enzymatically active and inactive EAL domains. J. Bacteriol..

[62] Ryjenkov DA, Tarutina M, Moskvin OV, Gomelsky M (2005). Cyclic diguanylate is a ubiquitous signaling molecule in bacteria: insights into biochemistry of the GGDEF protein domain. J. Bacteriol..

[63] Seidel SA (2013). Microscale thermophoresis quantifies biomolecular interactions under previously challenging conditions. Methods.

