# Supplementary material for: The Wsp chemosensory system modulates c-di-GMP-dependent biofilm formation by integrating DSF quorum sensing through the WspR-RpfG complex in Lysobacter
Source: NPJ Biofilms Microbiomes. 2022 Dec 16;8:97. doi: 10.1038/s41522-022-00365-1 (PMC9758175; doi:10.1038/s41522-022-00365-1)
Supplement: Supplementary file 1 — Supporting information [file 41522_2022_365_MOESM1_ESM.pdf]

Supplementary information

The Wsp chemosensory system modulates c-di-GMP-dependent biofilm formation by integrating DSF quorum sensing through the WspR-RpfG complex in *Lysobacter*

Kangwen Xu[a,c], Limin Wang[a], Dan Xiong[a], Hongjun Chen[b], Xinru Tong[b], Xiaolong Shao[a], Tao Li [b]*, Guoliang Qian[a]*

Content: 2 Supplementary Tables, 13 Supplementary Figures.

Supplementary Table 1. Strains and plasmids used in this study

**Strains and plasmids**	**Characteristics**[a]	**Source**
Wild-type strains		
Lysobacter enzymogenes OH11	Wild type, Km^R	1
In-frame deletion mutants		
Δ*wspA*	In-frame deletion of *wspA* in strain OH11, Km^R	2
Δ*wspB*	In-frame deletion of *wspB* in strain OH11, Km^R	2
Δ*wspC*	In-frame deletion of *wspC* in strain OH11, Km^R	2
Δ*wspD*	In-frame deletion of *wspD* in strain OH11, Km^R	2
Δ*wspE*	In-frame deletion of *wspE* in strain OH11, Km^R	2
Δ*wspF*	In-frame deletion of *wspF* in strain OH11, Km^R	3
Δ*wspR*	In-frame deletion of *wspR* in strain OH11, Km^R	2
Δ*lchP*	In-frame deletion of *lchP* in strain OH11, Km^R	4
Δ*rpfG*	In-frame deletion of *rpfG* in strain OH11, Km^R	4
Δ*wspF*-*wspF*^{D53A}	Chromosomal mutation of D53→A53 of WspF in wild-type, Km^R	This study
Δ*wspF*-*wspF*^{D53E}	Chromosomal mutation of D53→E53 of WspF in wild-type, Km^R	This study
Δ*wspF*-*wspF*^{S155A}	Chromosomal mutation of S115→A115 of WspF in wild-type, Km^R	2
Δ*wspF*-*wspF*^{H182A}	Chromosomal mutation of H182→A182 of WspF in wild type, Km^R	2
Δ*wspF*Δ*wspR*-*wspR*^{D72A}	In-frame deletion of *wspF* and chromosomal D72A mutation in the *wspR* gene, Km^R	2
Δ*wspF*Δ*wspR*-*wspR*^{GGAAF}	Chromosomal mutation of GGEEF→GGAAF of WspR in the *wspF* mutation, Km^R	2
Δ*wspA*Δ*rpfF*	Both in-frame deletion of *wspA* and *rpfF* in strain OH11, Km^R	This study
Δ*wspR*Δ*rpfF*	Both in-frame deletion of *wspR* and *rpfF* in strain OH11, Km^R	This study
Δ*wspA*Δ*wspF*	Both in-frame deletion of *wspA* and *wspF* in strain OH11, Km^R	2
Δ*wspE*Δ*wspF*	Both in-frame deletion of *wspE* and *wspF* in strain OH11, Km^R	2
Δ*wspF*Δ*wspR*	Both in-frame deletion of *wspF* and *wspR* in strain OH11, Km^R	2
Δ*wspF*Δ0901	Both in-frame deletion of *wspF* and *0901* in strain OH11, Km^R	2
Δ*wspF*Δ*lchD*	Both in-frame deletion of *wspF* and *3756* in strain OH11, Km^R	2

Complementary and overexpression strains		
Δ*wspF*(pBBR)	Δ*wspF* harbouring plasmid pBBR, Km^R, Gm^R	This study
Δ*wspF*(*yhjH*)	Δ*wspF* harbouring plasmid pBBR-*yhjH*, Km^R, Gm^R	This study
Δ*wspF*(GFP)	Δ*wspF* harbouring plasmid pBBR-GFP, Km^R, Gm^R	This study
Δ*rpfG*(GFP)	Δ*rpfG* harbouring plasmid pBBR-GFP, Km^R, Gm^R	This study
Δ*rpfG*(*rpfG*)	Δ*rpfG* harbouring plasmid pBBR-*rpfG*, Km^R, Gm^R	5
Δ*rpfG*(*rpfG*^{H190A})	Δ*rpfG* harbouring plasmid pBBR- *rpfG*^{H190A}, Km^R, Gm^R	5
Δ*rpfG*(*rpfG*^{Y254A})	Δ*rpfG* harbouring plasmid pBBR- *rpfG*^{Y254A}, Km^R, Gm^R	5
OH11(*wspR*)	OH11 harbouring plasmid pBBR-*wspR*, Km^R, Gm^R	2
OH11(*wspR*^{GGAAF})	OH11 harbouring plasmid pBBR-*wspR*$^{GGEEF \rightarrow GGAAF}$, Km^R , Gm^R	2
OH11(*wspC*)	OH11 harbouring plasmid pBBR-*wspC*, Km^R, Gm^R	2
OH11(*lchD*)	OH11 harbouring plasmid pBBR-*lchD*, Km^R, Gm^R	This study
Escherichia coli		
DH5α	Host strain for molecular cloning	6
XL1-Blue MRF' Kan	Host strain for bacterial two-hybrid assay	7
BL21(DE3)	Host strain for protein expression	8
Plasmids		
pEX18GM	Suicide vector with a *sacB* gene, Gm^R	9
pBBR1-MCS5	Broad-host- vector with a P_{lac} promoter, Gm^R	10
pTRG	The plasmid used for protein expression in bacterial two-hybridization assay, Tet^R	7
pBT	The plasmid used for protein expression in bacterial two-hybridization assay, $Chlo^R$	11
pET30a	Protein expression vector with a His tag, Km^R	8
pEX18-WspA	pEX18GM with two flanking fragments of *wspA*, Gm^R	2
pEX18-WspR	pEX18GM with two flanking fragments of *wspR*, Gm^R	2
pEX18-*wspF*^{D53A}	pEX18GM with fragment containing coding region of *wspF*^{D53A} and its two flanking fragments of *wspF*, Gm^R	This study
pEX18-*wspF*^{D53E}	pEX18GM with fragment containing coding region of *wspF*^{D53E} and its two flanking fragments of *wspF*, Gm^R	This study
pBBR-*yhjH*	pBBR1-MCS5 with fragment *yhjH*, Gm^R	4
pET30a-WspR	pET30a with the coding region of *wspR*, Km^R	2
pET30a-WspR$^{GGEEF \rightarrow GGAAF}$	pET30a with the coding region of *wspR*^{GGAAF}, Km^R	2
pET30a-WspR$^{\Delta REC}$	pET30a with the coding region of *wspR*$^{\Delta REC}$, Km^R	2
pET30a-RpfG-FLAG	pET30a containing the coding region of *rpfG* with C-FLAG	This study
pET30a-HD-GYP-FLAG	pET30a containing the coding region of HD-GYP domain of *rpfG* with C-FLAG	This study
pMAL-p2x	Protein expression vector with a MBP tag, Amp^R	5
pMAL-RpfG	pMAL with the coding region of *rpfG*, Amp^R	5
pMAL-RpfG^{H190A}	pMAL with the coding region of *rpfG*^{H190A}, Amp^R	5
pTRG-WspR	pTRG with the coding region of *wspR* from strain OH11, Tet^R	2
pBT-RpfG	pBT with the coding region of *rpfG* from strain OH11, Chl^R	This study

[a]Km^R, Gm^R Amp^R, Tet^R, $Chlo^R$, kanamycin, gentamicin, ampicillin, tetracycline, chloramphenicol resistance, respectively.

Supplementary Table 2. Primers used in this study

**Primer**	**Sequence (5’-3’)** [a]	**Use**
Point mutation		
wspF^{D53A}-F1	GGGGTACCTGGGCAAGGTCAAGGACATC(*Kpn*I)	To amplify a 760-bp upstream of *wspF*^{D53A}
wspF^{D53A}-R1	CGTTCATGCCGGGCATGACCAGGGCCATCAGCACCA GGTCGG	
wspF^{D53A}-F2	CCGACCTGGTGCTGATGGCCCTGGTCATGCCCGGCAT GAACG	To amplify a 1362-bp downstream of *wspF*^{D53A}
wspF^{D53A}-R2	CCCAAGCTTATCAGGCGTTTCTGGTGCTC(*Hind*III)	
Protein expression		
rpfG-FLAG-F	GGAATTCCATATGCTGCGCCACATCATCGA GGACATTG (*Nde*I)	To amplify a 1041-bp fragment containing coding region of *rpfG* fused FLAG tag
rpfG-FLAG-R	CCCAAGCTTTTACTTGTCATCGTCGTCCTTGT AATCCTCCAGGCCGGGCCTGCTGGGTAC (*Hind*III)	
HD-GYP-FLAG-F	GGAATTCCATATGCGCGACGCCGGCACCAGC GCCTACCTGGAGCGC (*Nde*I)	To amplify a 414-bp fragment containing coding region of HD-GYP domain of *rpfG* fused FLAG tag
HD-GYP-FLAG-R	CCCAAGCTTTTACTTGTCATCGTCGTCCTTGT AATCGGCCTTCTTGTACGGACGCGGCG (*Hind*III)	
bacterial two hybrid		
pBT-*rpfG*-F	GAAGAGACGTTTGGCGCGGCCGCAAT GCTGCGCCACATCATCGAGGACATTG	To amplify a 1017-bp fragment containing coding region of *rpfG*
pBT-*rpfG*-R	TTAACTCGAGGATCCCCGGGAATTCTTA CTCCAGGCCGGGCCTGCTGGGTAC	

[a] Restricted digestion enzyme site was underlined.

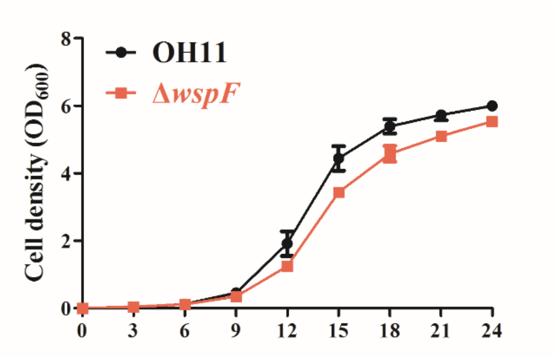

Supplementary Figure 1. Effects of WspF on the growth ability of *L. enzymogenes* OH11 in LB broth.

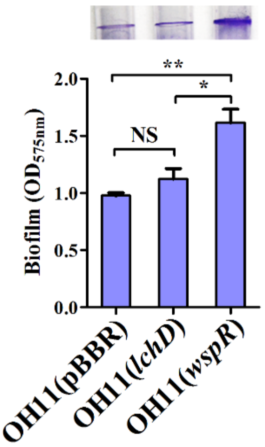

Supplementary Figure 2. Biofilm formation in *wspR* and *lchD* overexpressing strains. OH11, wild-type strain. pBBR, short for pBBR1-MCS5, is a vector control.

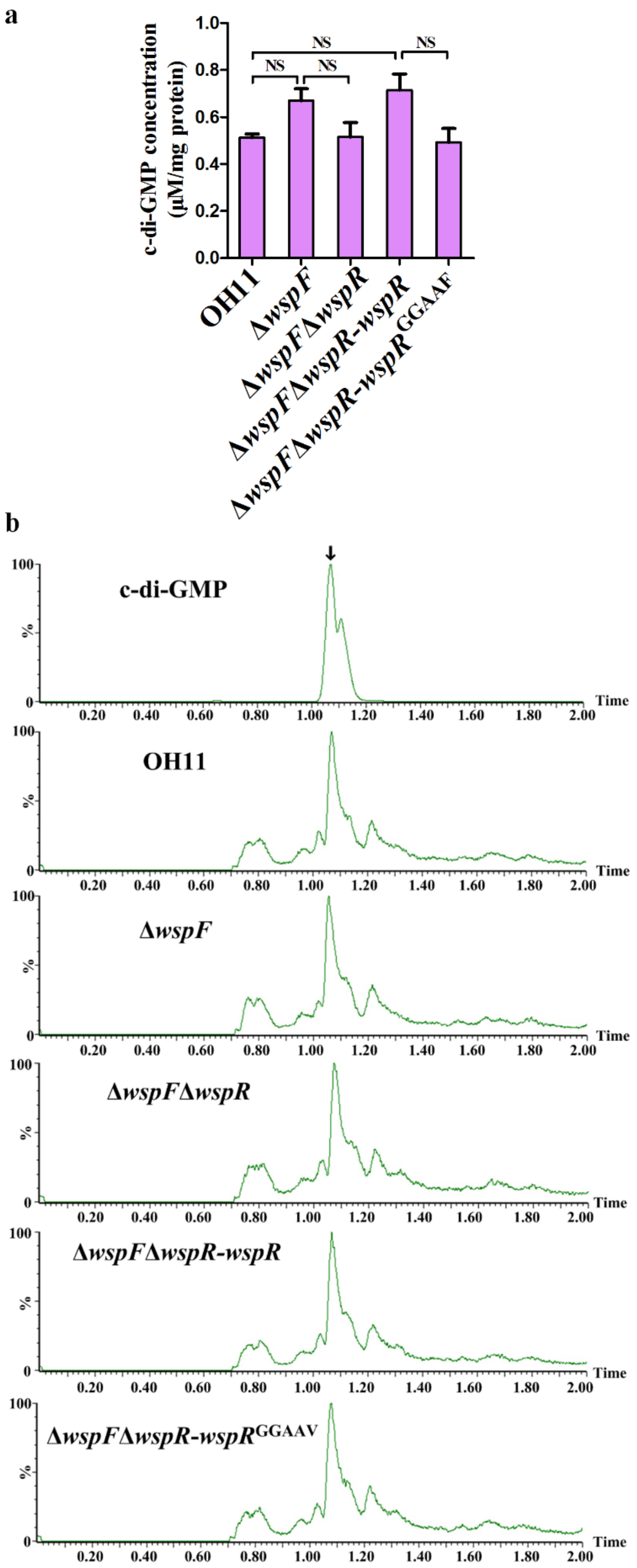

Supplementary Figure 3. Total intracellular c-di-GMP concentration determined by LC-MS/MS. a The total c-di-GMP level of all tested strains remained consistent with the wild type. **b** LC-MS/MS chromatogram corresponding to **a**. The black arrow represents the peak time of c-di-GMP. Statistical comparisons were performed using

one-way ANOVA of GraphPad software (GraphPad, La Jolla, CA). In panel **a**, the mean data of ±SD from three experiments were shown, $**P < 0.01$. NS means not significant.

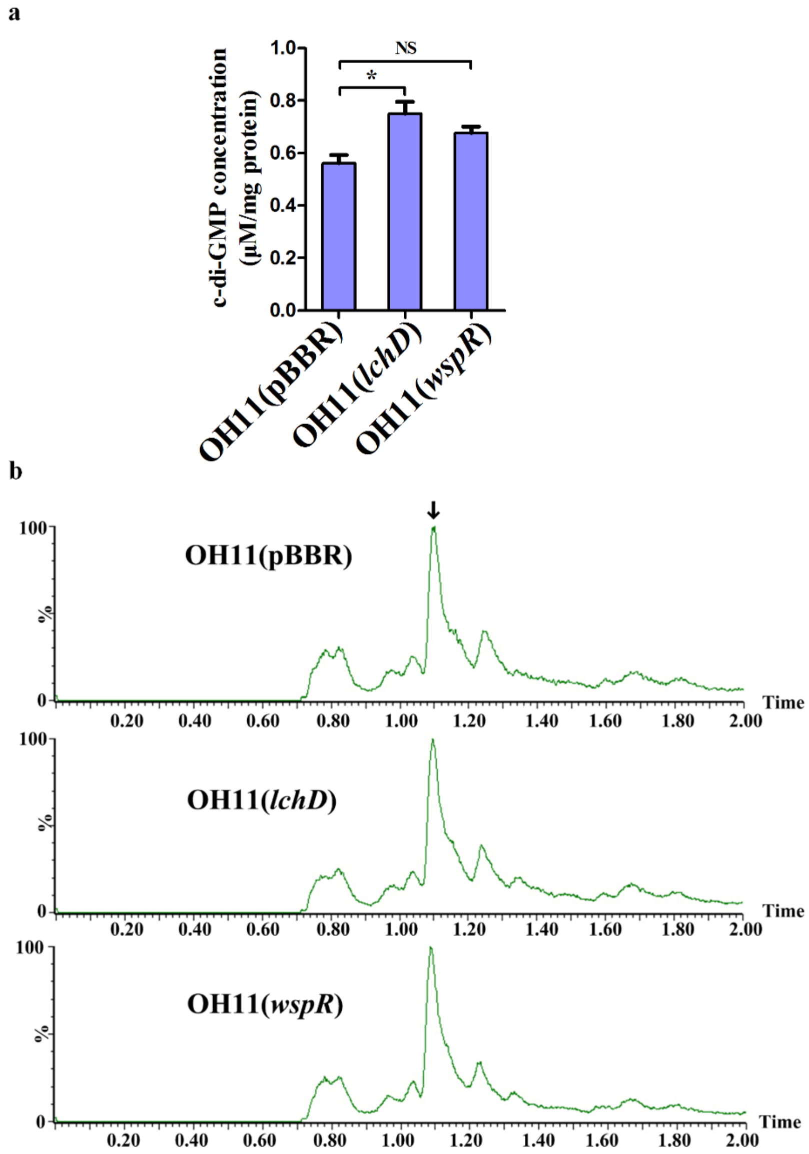

Supplementary Figure 4. Total c-di-GMP level of *lchD* and *wspR* overexpressing strains determined by LC-MS/MS. a The total c-di-GMP level of *lchD* overexpressing strains was modestly higher than that of the wild type, while the *wspR* overexpressing strains did not change the total content of c-di-GMP. **b** LC-MS/MS chromatogram corresponding to **a**. The black arrow represents the peak time of c-di-GMP. Statistical comparisons were performed with using one-way ANOVA of GraphPad software (GraphPad, La Jolla, CA). In panel **a**, mean data ±SD from three experiments were shown, $*P < 0.05$. NS means not significant.

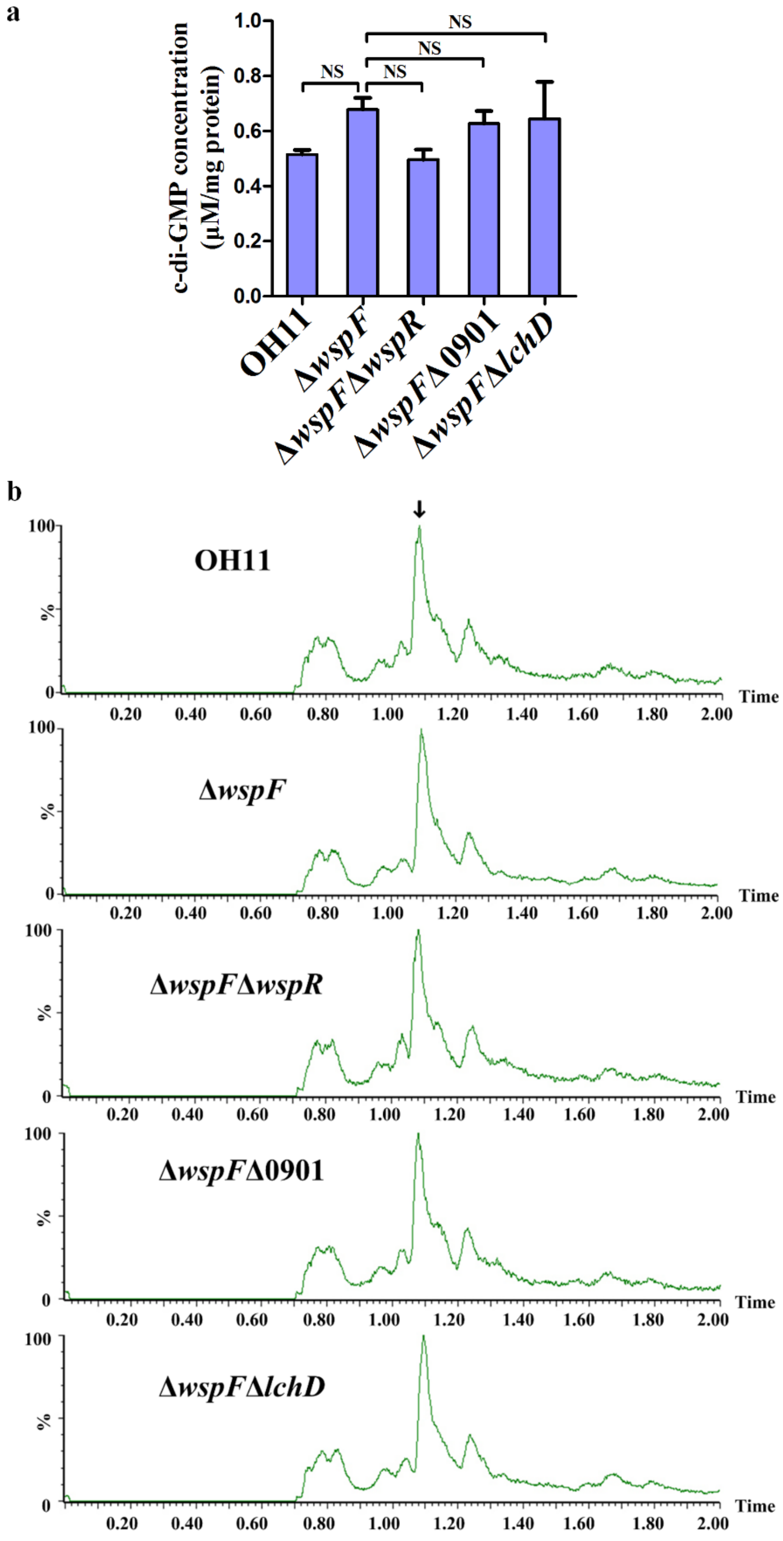

Supplementary Figure 5. Total c-di-GMP level of *wspF*0901 and *wspFlchD* double mutants determined by LC-MS/MS. a The total c-di-GMP level of all tested strains remained consistent with the wild type. **b** LC-MS/MS chromatogram corresponding to **a**. The black arrow represents the peak time of c-di-GMP. Statistical comparisons were performed using one-way ANOVA of GraphPad software (GraphPad, La Jolla, CA). In

panel **a**, mean data ±SD from three experiments were shown, ***P* < 0.01. NS means not significant.

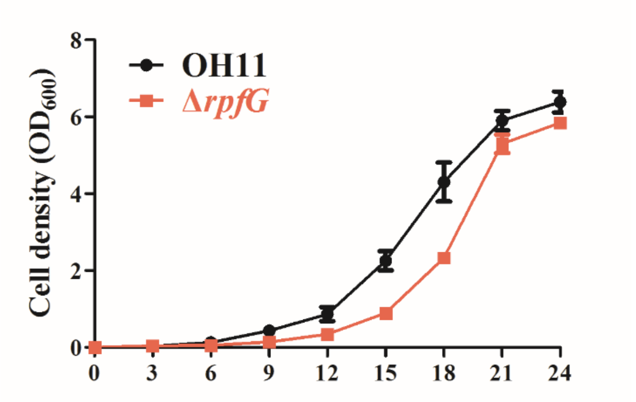

Supplementary Figure 6. Effects of RpfG on the growth ability of *L. enzymogenes* OH11 in LB broth.

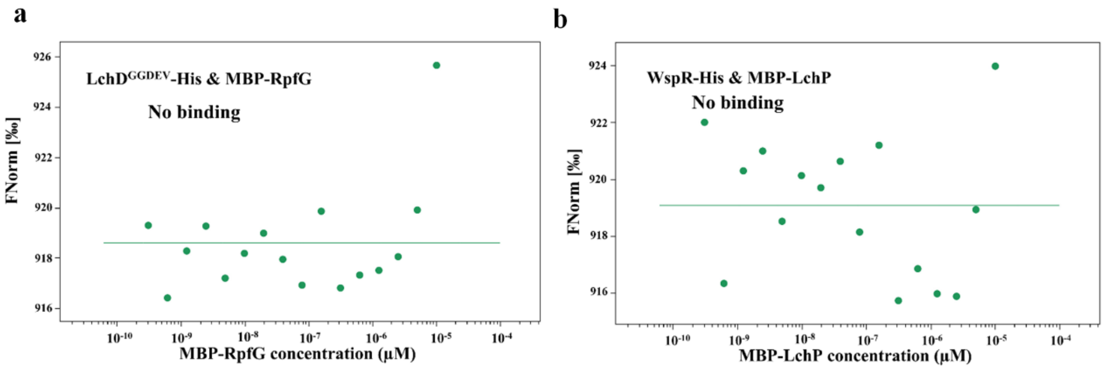

Supplementary Figure 7. MST analysis showed that the intracellular portion of LchD (LchD^{GGDEV}-His) did not interact with RpfG (a), and WspR did not interact with MBP-LchP (b).

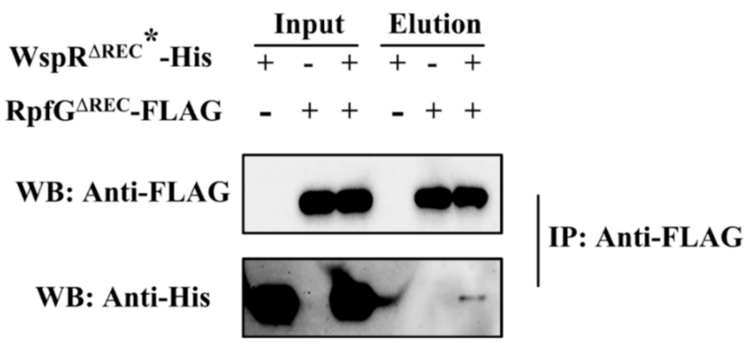

Supplementary Figure 8. The HD-GYP domain of RpfG interacts with the enzymatically inactive GGDEF domain of WspR. A pull-down assay confirming the direct interaction of the HD-GYP domain of RpfG (RpfG$^{\Delta REC}$) with the enzymatically

inactive GGDEF domain of WspR (WspR$^{\Delta REC*}$) by changing the "GGDEF" motif to "GGAAF". IP assay was performed using anti-FLAG antibody. Western blotting was performed using anti-FLAG and anti-His antibodies. All blots derive from the same experiment and that they were processed in parallel.

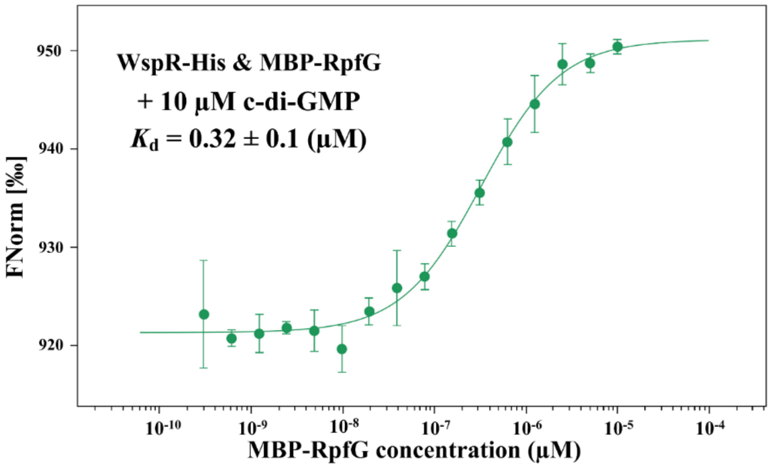

Supplementary Figure 9. The physiological range of c-di-GMP does not affect WspR-RpfG binding. MST showed that WspR-His still interacted with MBP-RpfG with moderate affinity (K_d, 0.32 µM) in the presence of 10-µM c-di-GMP.

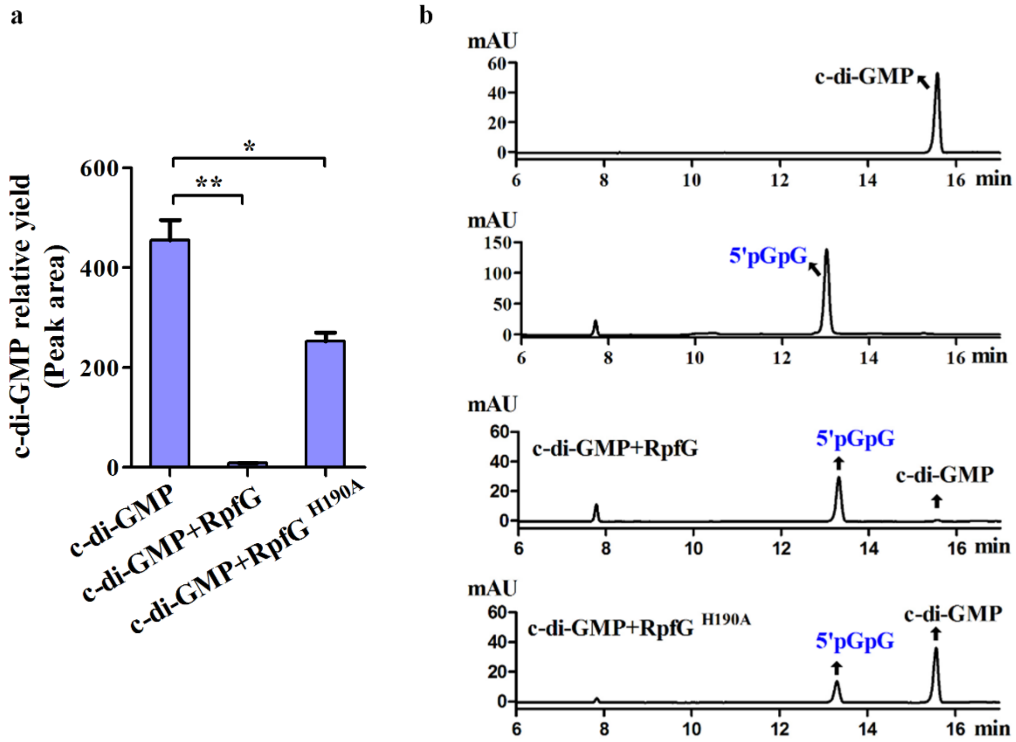

Supplementary Figure 10. RpfG is active in degrading c-di-GMP *in vitro* as

detected by HPLC. a Purified MBP-RpfG is highly active while MBP-RpfG^{H190A} is moderately active against c-di-GMP *in vitro*. **b** HPLC chromatogram corresponding to **a**. The c-di-GMP and 5'pGpG standard are shown in black and blue, respectively. Statistical comparisons were performed using one-way ANOVA of GraphPad software (GraphPad, La Jolla, CA). In panels **a**, mean data ±SD from three experiments were shown, **$P < 0.01$.

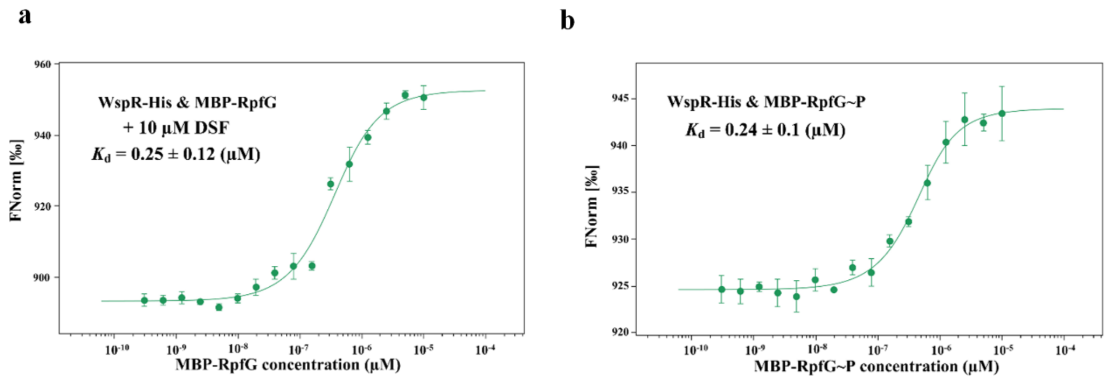

Supplementary Figure 11. DSF or RpfG phosphorylation did not affect WspR-RpfG binding. a MST showed that WspR-His still interacted with MBP-RpfG with moderate affinity (K_d, 0.25 µM) in the presence of 10-µM DSF. **b** MST showed that RpfG phosphorylation still interacted with WspR-His with moderate affinity (K_d, 0.24 µM).

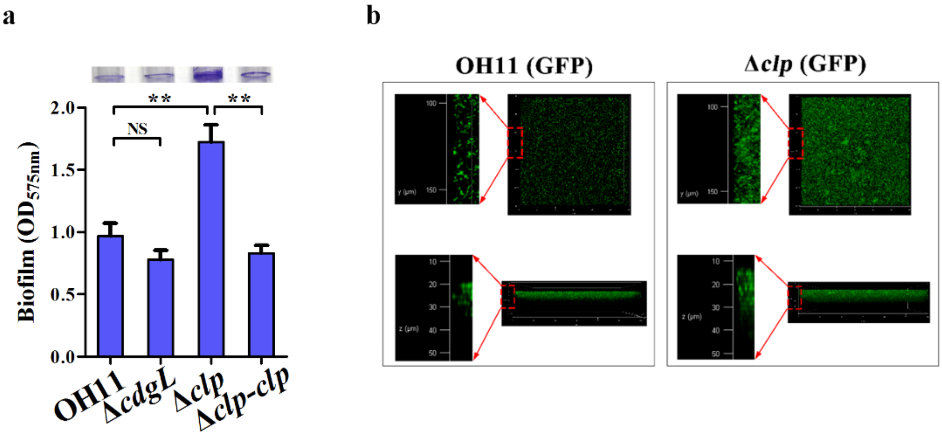

Supplementary Figure 12. Clp, a well-known c-di-GMP-binding transcription factor, negatively regulated biofilm formation in *L. enzymogenes*. a Clp, a c-di-GMP receptor, but not CdgL, positively regulated biofilm formation. Δ*clp-clp*, the native *clp*, was chromosomally inserted into the *clp* mutant. **b** CLSM of biofilms formed by wild-

type and *clp* mutants. The top faces (y and x axis) of the three-dimensional biofilm structure formed by the tested strains was shown in the upper part of the black box. The side faces (z and x axis) of the three-dimensional biofilm structures formed by the test strains were shown in the lower part of the black box. In each section, an enlarged view of the red circle was presented on the left. Statistical comparisons were performed using one-way ANOVA of GraphPad software (GraphPad, La Jolla, CA). In panels **a**, mean data ±SD from three experiments were shown, $^{**}P < 0.01$. NS means not significant.

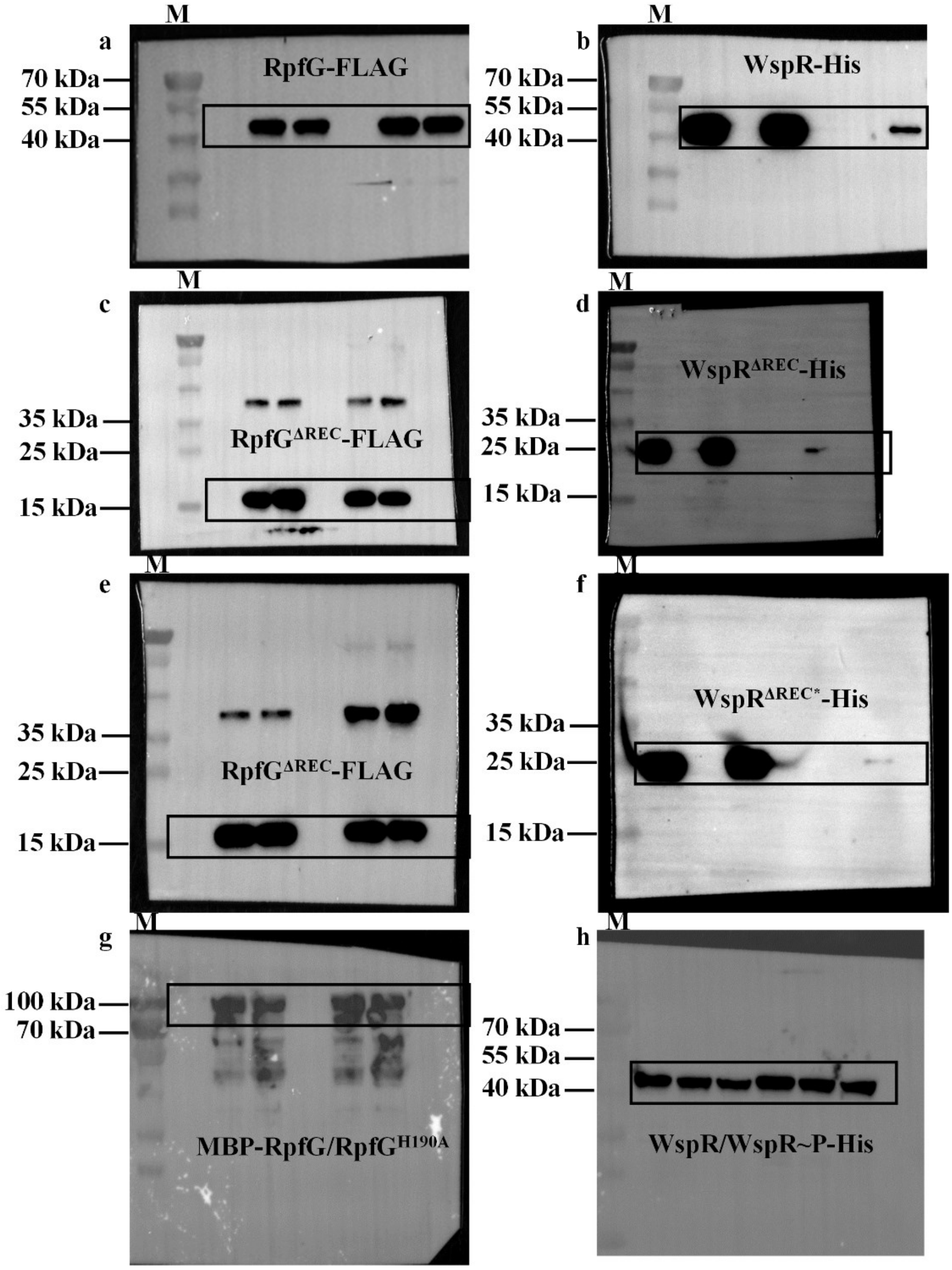

Supplementary Figure 13. Uncropped gels & blots. a&b Box highlighted by black

corresponds to Fig. 4b. **c&d** Box highlighted by black corresponds to Fig. 4f. **e&f** Box highlighted by black corresponds to Supplementary Fig. 8. **g&h** Box highlighted by black corresponds to Fig. 5f & 6e.

Supplementary References

1 Qian, G., Hu, B., Jiang, Y. & Liu, F. Identification and characterization of *Lysobacter enzymogenes* as a biological control agent against some fungal pathogens. Agric. Sci. China. **8**, 68-75 (2009).

2 Xu, K. et al. A non-flagellated, predatory soil bacterium reprograms a chemosensory system to control antifungal antibiotic production via cyclic di-GMP signalling. Environ. Microbiol. **23**, 878-892 (2021).

3 Chen, Y. et al. *Lysobacter* PilR, the regulator of type IV pilus synthesis, controls antifungal antibiotic production via a cyclic di-GMP pathway. Appl. Environ. Microb. **83**, e03397-16 (2017).

4 Xu, G. et al. Signaling specificity in the c-di-GMP-dependent network regulating antibiotic synthesis in *Lysobacter*. Nucleic. Acids. Res. **46**, 9276-9288 (2018).

5 Li, K. et al. The predatory soil bacterium *Lysobacter* reprograms quorum sensing system to regulate antifungal antibiotic production in a cyclic-di-GMP-independent manner. Commun. Biol. **4**, 1131 (2021).

6 Qian, G. et al. *Lysobacter enzymogenes* uses twodistinct cell-cell signaling systems for differential regulation of secondary-metabolite biosynthes isand colony morphology. Appl. Environ. Microb. **79**, 6604-6616 (2013).

7 Guo, M. et al. Dissecting transcription regulatory pathways through a new bacterial one-hybrid reporter system. Genome. Res. **19**, 1301-1308 (2009).

8 Su, Z. et al. 4-Hydroxybenzoic acid is a diffusible factor that connects metabolic shikimate pathway to the biosynthesis of a unique antifungal metabolite in *Lysobacter enzymogenes*. Mol. Microbiol. **104**, 163-178 (2017).

9 Hoang, T T., Karkhoff-Schweizer, R. R., Kutchma, A.J. & Schweizer, H. P. A broad-host-range Flp-*FRT* recombination system for site-specific excision of chromosomally-located DNA sequences: application for isolation of unmarked *Pseudomonas aeruginosa* mutants. Gene **212**, 77-78 (1998).

10 Kovach, M. E. et al. Four new derivatives of the broad-host-range cloning vector pBBR1MCS, carrying different antibiotic-resistance cassettes. Gene **166**, 175-176 (1995).

11 Fang, X. et al. GIL, a new c-di-GMP-binding protein domain involved in regulation of cellulose synthesis in *enterobacteria*. Mol. Microbiol. **93**, 439-452 (2014).
